# A Dominant-Negative Mutation of Mouse *Lmx1b* Causes Glaucoma and Is Semi-lethal via LBD1-Mediated Dimerisation

**DOI:** 10.1371/journal.pgen.1004359

**Published:** 2014-05-08

**Authors:** Sally H. Cross, Danilo G. Macalinao, Lisa McKie, Lorraine Rose, Alison L. Kearney, Joe Rainger, Caroline Thaung, Margaret Keighren, Shalini Jadeja, Katrine West, Stephen C. Kneeland, Richard S. Smith, Gareth R. Howell, Fiona Young, Morag Robertson, Rob van t' Hof, Simon W. M. John, Ian J. Jackson

**Affiliations:** 1 MRC Human Genetics Unit, MRC IGMM, University of Edinburgh, Edinburgh, United Kingdom; 2 The Howard Hughes Medical Institute, The Jackson Laboratory, Bar Harbor, Maine, United States of America; 3 Centre for Genomic and Experimental Medicine, IGMM, Edinburgh, United Kingdom; 4 UCL Institute of Ophthalmology, London, United Kingdom; 5 Electron Microscopy, Pathology, Western General Hospital, Edinburgh, United Kingdom; 6 The Roslin Institute, University of Edinburgh, Easter Bush, United Kingdom; Stanford University School of Medicine, United States of America

## Abstract

Mutations in the LIM-homeodomain transcription factor *LMX1B* cause nail-patella syndrome, an autosomal dominant pleiotrophic human disorder in which nail, patella and elbow dysplasia is associated with other skeletal abnormalities and variably nephropathy and glaucoma. It is thought to be a haploinsufficient disorder. Studies in the mouse have shown that during development *Lmx1b* controls limb dorsal-ventral patterning and is also required for kidney and eye development, midbrain-hindbrain boundary establishment and the specification of specific neuronal subtypes. Mice completely deficient for *Lmx1b* die at birth. In contrast to the situation in humans, heterozygous null mice do not have a mutant phenotype. Here we report a novel mouse mutant *Icst*, an *N*-ethyl-*N*-nitrosourea-induced missense substitution, V265D, in the homeodomain of LMX1B that abolishes DNA binding and thereby the ability to transactivate other genes. Although the homozygous phenotypic consequences of *Icst* and the null allele of *Lmx1b* are the same, heterozygous *Icst* elicits a phenotype whilst the null allele does not. Heterozygous *Icst* causes glaucomatous eye defects and is semi-lethal, probably due to kidney failure. We show that the null phenotype is rescued more effectively by an *Lmx1b* transgene than is *Icst*. Co-immunoprecipitation experiments show that both wild-type and *Icst* LMX1B are found in complexes with LIM domain binding protein 1 (LDB1), resulting in lower levels of functional LMX1B in *Icst* heterozygotes than null heterozygotes. We conclude that *Icst* is a dominant-negative allele of *Lmx1b*. These findings indicate a reassessment of whether nail-patella syndrome is always haploinsufficient. Furthermore, *Icst* is a rare example of a model of human glaucoma caused by mutation of the same gene in humans and mice.

## Author Summary

Nail-patella syndrome is a human genetic disease caused by an inactivating mutation in one copy of a gene called *LMX1B*, with the amount of protein produced from the remaining copy of the gene not being enough for normal function. Patients with this disease have malformations of their nails, elbows and kneecaps. Some patients also develop kidney disease and glaucoma. *LMX1B* controls where and when other genes are expressed and it is important during development. Studies in mice have shown that complete absence of *Lmx1b* is lethal at birth. In contrast to humans, mice with only one copy of the gene are normal. Here we describe a new mutant mouse, *Icst*, which has a mutation in *Lmx1b* that abolishes the ability of the protein to bind near genes that it controls. Mice with one normal and one copy of *Lmx1b* with the *Icst* mutation have eye defects and some die shortly after birth probably due to kidney failure. Therefore having one functional and one mutant copy of *Lmx1b* is more detrimental than having a half dose of functional protein. The *Icst* mouse is a model of human glaucoma where mutation of the same gene causes glaucoma in humans and mice.

## Introduction

Nail-patella syndrome (NPS) (OMIM 161200) is an autosomal dominant human disease, cardinal features of which are nail dysplasia, absent or hypoplastic patellae and abnormal elbows along with iliac horns. In addition, about 30–40% of patients develop nephropathy, which can progress to renal disease [1]. Open angle glaucoma is another feature of the disease that occurs in about 30–40% of patients [2]. Mutations of the transcription factor *LMX1B* have been found to be the underlying cause of NPS [3]–[6]. LMX1B is a member of the LIM-homeodomain (LIM-HD) family of transcription factors. The protein has two N-terminal LIM domains that are involved in protein-protein interactions followed by a homeodomain that binds to target DNA binding sites. Disease-causing mutations range from complete gene deletion to various frameshift, nonsense, splice and missense mutations. The majority of missense mutations are found in the homeodomain and the LIM domains. There is great variation in the severity and range of phenotypes both within families that carry the same mutation and between families that carry different mutations in *LMX1B*. Several missense NPS homeodomain mutations tested *in vitro* have shown no dominant-negative effect on the transcriptional activity of wild-type protein [7], [8] and consequently NPS is thought to be a haploinsufficient disorder. Nevertheless, in a comprehensive study of 106 NPS patients from 32 families, patients with mutations in the homeodomain had more severe proteinurea than those with mutations of the LIM domains, although other aspects of NPS showed no phenotype-genotype correlation [9]. Association of haplotype with severity of nail dysplasia has also been reported [10].

Knockout studies in mice have shown that *Lmx1b* is required during development for dorsal patterning of the limb, the establishment of the midbrain-hindbrain boundary, the development of the cerebellum, for kidney development and for the specification of certain neuronal subtypes (reviewed in [11]). Mice that lack *Lmx1b* have ventralised limbs, kidney abnormalities, calvarial bone defects and an absent cerebellum [12]–[14]. There are also anterior segment eye defects [15]. Postnatal conditional deletion experiments have shown that *Lmx1b* is required for formation of the trabecular meshwork, the maintenance of corneal integrity and transparency and loss results in corneal neovascularisation [16]. Heterozygous knockout mice are apparently normal indicating that in the mouse haploinsufficiency for *Lmx1b* does not lead to mutant phenotypes [12], [15]. However, heterozygous knockout mice recover less well from unilateral nephrectomy than wild-type mice, suggesting some role in adult kidney regeneration and maintenance [17].

Here we report the identification of an *Lmx1b* missense mutation, *Icst*, which has a dominant-negative mode of action. In contrast to heterozygous knockout mice, heterozygous *Lmx1b^Icst^* mice have buphthalmic (enlarged or bulging) eyes and develop a glaucoma phenotype. In addition there is some post-natal lethality associated with kidney defects. We demonstrate that the difference in phenotypic consequence of the null and *Icst* alleles is due to LMX1B^Icst^ protein acting in a dominant-negative manner. These findings have implications for the interpretation of the mode of action of mutant LMX1B in NPS.

## Results

### *Icst* is a missense mutation of the *Lmx1b* gene

**Figure 1:**
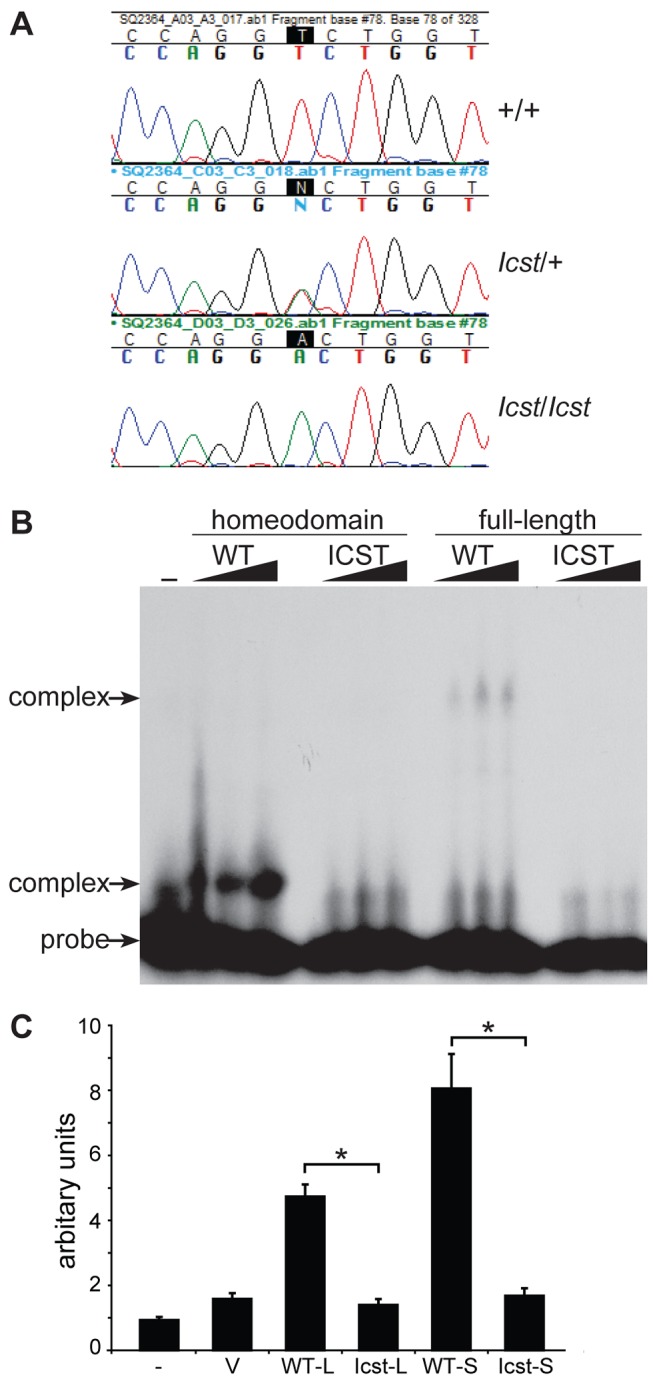
*Icst* is a homeodomain missense mutation of *Lmx1b* that abolishes protein function. (A) Genomic DNA sequence traces from *Lmx1b* exon 5 from wild-type (WT), heterozygous mutant (*Icst*/+) and homozygous mutant (*Icst*/*Icst*) embryonic samples. The position of the T-to-A transversion at position 725 (725T>A) in the *Lmx1b* gene, numbering from Genbank entry AF078166, is highlighted. This transversion causes a V265D mutation in the homeodomain numbering from entry O88609 in http://www.uniprot.org. (B) The *Icst* mutation impairs binding of LMX1B. Bandshift analysis using a fixed amount of ^32^P-labelled FLAT LMX1B binding element [21] with increasing amounts (as indicated by the triangles) of His-tagged fusion proteins containing the homeodomain or full-length LMX1B either wild-type (WT) or containing the *Icst* mutation (ICST). In lane 1 no protein was added (-). Complexes as indicated by the arrows were formed with the wild-type proteins but not the proteins containing the *Icst* mutation. The position of the probe alone is also indicated by an arrow. (C) The *Icst* mutation abolishes transcriptional activity. A luciferase reporter plasmid, 6×FLAT-C2P-Luc4, containing six copies of the LMX1B binding element FLAT upstream of the *Col2a1* promoter ([21], kind gift of Brendan Lee), was either transfected alone (-) or co-transfected with empty pcDNA-3.1 vector (V) or pcDNA-3.1 containing the wild-type (WT-L) or Icst (Icst-L) long form of LMX1B or wild-type (WT-S) or Icst (Icst-S) short form of LMX1B. Each experiment was performed in triplicate, and mean firefly luciferase expression normalized to control renilla luciferase expression is shown+standard error. Both WT-L and WT-S elicit robust induction of luciferase expression that reduced to background levels by the *Icst* mutation (t-test; * = P<0.005).

We identified the *N*-ethyl-*N*-nitrosourea-induced mouse mutation, iris-corneal strands (*Icst*), in a screen for dominant eye mutations. Mice that carry *Icst* have buphthalmic eyes suggestive of high intra-ocular pressure [18]. We had previously mapped *Icst* to proximal Chr 2 between the markers *D2Mit365* and *D2Mit372* [18]. Within this interval we considered *Lmx1b* to be a strong candidate for harbouring the *Icst* mutation because glaucoma occurs in about 30–40% of NPS patients [2]. We amplified and sequenced the exons and flanking regions of *Lmx1b* from *Icst* mutant mice and found a single nucleotide change, a T to A transversion in exon 5 in the *Icst* allele (position 2:33,566,910 in Ensembl Release 74 mouse assembly GRCm38 (http://www.ensembl.org)) which was absent from the reference mouse sequence and an additional 17 mouse strains [19] (Figure 1A). This mutation causes substitution of hydrophobic valine with hydrophilic aspartic acid in the homeodomain (V265D). The affected valine is in the recognition helix, and is very highly conserved across species and paralogues. In the crystal structure of the related paired-type homeodomain, the equivalent valine directly contacts the DNA recognition sequence by making hydrophobic contacts with the second thymine in the TAAT core of the recognition sequence [20]. The nature of the mutation in *Lmx1b*, coupled with the complementation tests described below, indicate that *Icst* is the causative mutant allele; *Lmx1b^Icst^*. Examination of the sequence traces of RT-PCR products spanning exon 5 of *Lmx1b* from embryonic samples indicated that equal amounts of mutant and wild-type transcript are produced in heterozygotes (data not shown).

We asked whether the mutant LMX1B^Icst^ protein was able to bind to its recognition sequence. We produced recombinant His-tagged full-length LMX1B protein (S form, see below) and the homeodomain alone, in both wild-type and *Icst* mutant versions, and used these in bandshift experiments to determine if they could bind to a known LMX1B recognition sequence from intron 1 of the *Col4a4* gene [21]. Both wild-type full-length and homeodomain proteins bound but the LMX1B^Icst^ proteins did not (Figure 1B). We then investigated the ability of wild-type and mutant LMX1B protein to transactivate transcription in a reporter assay. We tested two isoforms of the mouse LMX1B protein, a 372 amino acid protein (S) and a longer form (L) that includes an additional 29 N-terminal amino acids initiating from an upstream AUG (see Materials and Methods) [22]. Much of this additional sequence is conserved between humans and mouse but also includes a direct 18 bp repeat encoding a further six amino acids. We introduced the *Icst* mutation into the two isoforms and tested the ability of the wild-type and mutant versions to transactivate a luciferase reporter, previously shown to be activated by LMX1B, which has six copies of the LMX1B recognition sequence upstream of a minimal promoter [21]. Both L and S wild-type proteins transactivated the luciferase reporter construct on co-transfection, but neither of the LMX1B^Icst^ proteins showed any transactivation activity (Figure 1C). These results showed that the *Icst* mutation disrupts the ability of the LMX1B protein to recognise and bind to its target sequences and activate transcription.

### Heterozygous *Lmx1b^Icst^* eye phenotype is a model of glaucoma

**Figure 2:**
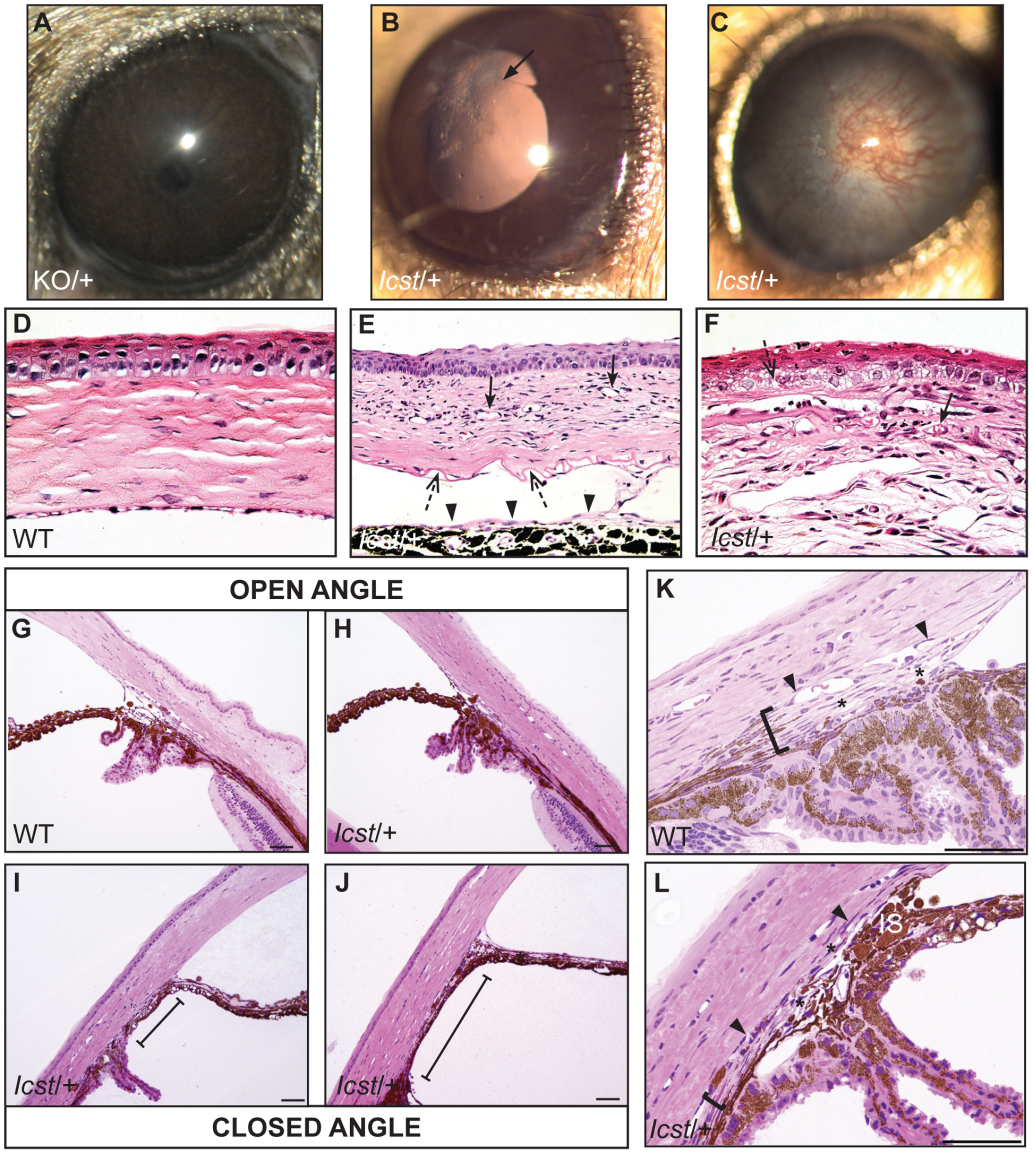
*Icst* causes defects in the anterior segment of the eye. (A) *Lmx1b^KO/+^* (KO/+) eye that is of normal appearance. (B) *Lmx1b^Icst/+^* (*Icst*/+) eye where there is a fine strand between the pupillary margin and the cornea (arrowed). (C) This *Lmx1b^Icst/+^* eye is enlarged with severe corneal vascularisation and central opacity. (D–F). Histological appearance of corneas. (D) Wild-type normal cornea (WT). (E) *Lmx1b^Icst/+^* cornea that has stromal vascularisation (barbed arrows). Descemet's membrane is wrinkled (dashed arrows) and a membrane is present on the anterior surface of the iris (arrowheads). (F) *Lmx1b^Icst/+^* cornea with basal epithelial oedema (dashed arrow). A stromal blood vessel with red blood cells is indicated by a barbed arrow. (G) Normal wild-type iridocorneal angle. (H–J) Iridocorneal angle morphology of *Lmx1b^Icst^* heterozygotes display a spectrum of phenotypes ranging from open-angle (H) to closed-angle morphology where the iris can be seen adhering to the cornea and blocking the iridocorneal angle as indicated by bars (I, J). (K–L) Higher magnification of wild-type (K) and *Lmx1b^Icst/+^* (L) iridocorneal angles. (K) The wild-type has an open-angle with robust drainage structures ([). Schlemm's canal (arrowheads) is clearly identifiable and the trabecular meshwork (asterisks) has well-formed trabecular beams. (L) The *Lmx1b^Icst/+^* iridocorneal angle is open but narrow. Iridocorneal strands (IS) are present. These are a normal feature of the mouse angle but are not present at all locations and do not prevent aqueous humor drainage. The drainage structures are not as robust as in the WT ([). Schlemm's canal is present but compressed (arrowheads) and the trabecular meshwork is hypoplastic (asterisks). Original magnification ×40 (D and F) and ×20 (E). Scale bar = 50 µm.

**Figure 3:**
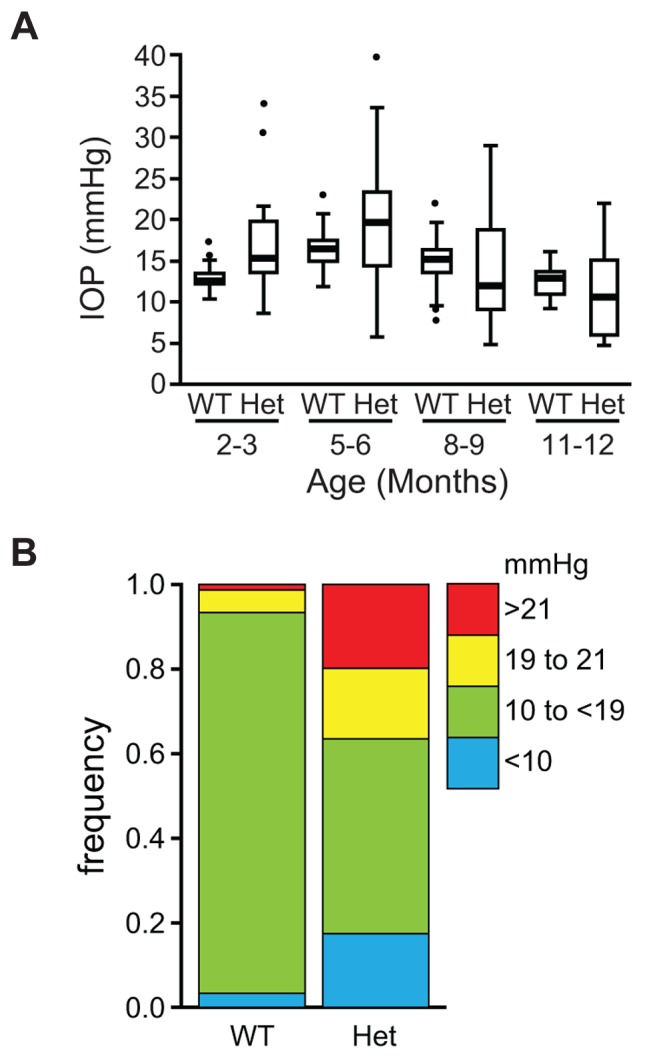
The IOP is elevated in *Lmx1b^Icst/+^* mice. (A) Box plot of IOP measurements of wild-type (WT) and *Lmx1b^Icst/+^* (Het) mice taken at different ages. There is significantly elevated IOP in the *Lmx1b^Icst/+^* mice up to 6 months (P = 0.006 at 2–3 months and P = 0.009 at 5–6 months). The IOP measurements are not significantly different in older mice owing to low readings of <10 mmHg in increasing numbers of the *Lmx1b^Icst/+^* mice. (B) Frequency histogram summarizing the data from panel A showing increased incidence of high IOP measurements in *Lmx1b^Icst/+^* mice.

**Figure 4:**
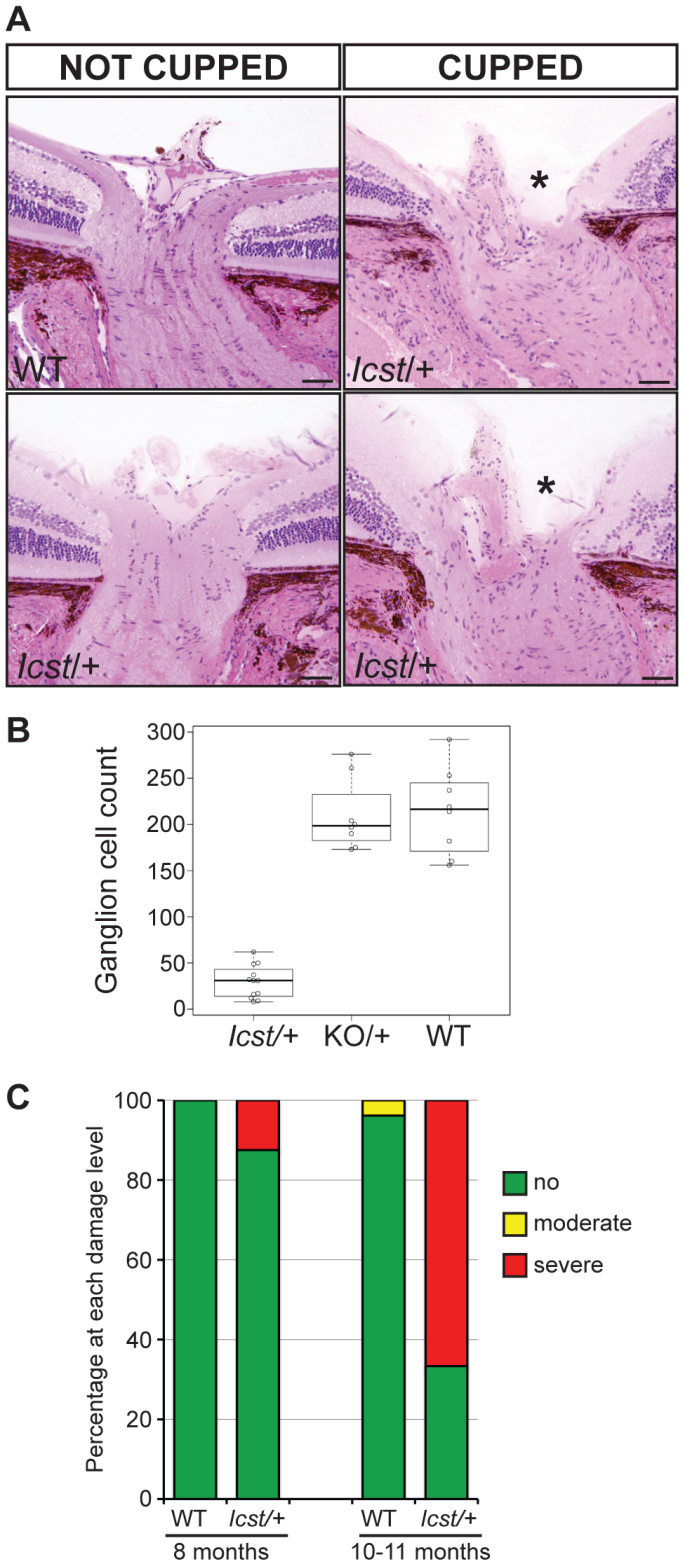
*Icst* causes defects of the optic nerve and the ganglion cell layer of the retina. (A) Sections through the optic nerve of adult mice. The optic nerve is variably affected in *Lmx1b^Icst/+^* mice. Wild-type (WT) is normal (upper panel, left). In some *Lmx1b^Icst/+^* (*Icst*/+) mice the optic nerve appears normal (lower panel, left) but in others there is evidence of optic nerve damage and cupping, indicated by asterisks (upper and lower panels, right). Scale bar = 50 µm. (B) Box plot of ganglion cell number present in *Lmx1b^Icst/+^* (*Icst*/+), *Lmx1b* ^KO/*+*^ (KO/+) and wild-type (WT) mice. Ganglion cells were stained using an anti-BRN3 antibody and the number present in each of four areas from around the optic disc from one eye were counted (n = 3 for *Lmx1b^Icst/+^* and n = 2 for the other two genotypes). Compared to wild-type and *Lmx1b* ^KO/*+*^ mice which show no difference (P = 0.95) the number of ganglion cells is greatly reduced in the *Lmx1b^Icst^* ^/*+*^ mice compared to wild-type (P = 0.017) and to *Lmx1b* ^KO/*+*^ mice (P = 0.034). (C) Optic nerve damage in *Lmx1b^Icst/+^* mice. Optic nerve damage was assessed in wild-type (WT) and *Lmx1b^Icst^* ^/*+*^ (*Icst*/+) mice at 8 months (WT, n = 23 and *Icst*/+, n = 16) and at 10–11 months (WT, n = 26 and *Icst*/+, n = 36). Nerves with no glaucoma have axon counts that match controls (green bar). Nerves with moderate nerve damage have an average of 30% axon (yellow bar). This degree of damage occurs in a low percentage of wild-type of this strain background at this age [59]. All severely affected nerves have extensive axon damage throughout the optic nerve with obvious axon loss ranging from 50% to almost complete axon loss (red bar).

Mice that are heterozygous for the *Lmx1b* knockout allele do not have an eye phenotype (Figure 2A) [15]. In contrast, we observed that the eyes of *Lmx1b^Icst/+^* mice had mild corneal opacity at a young age and by six to eight weeks *Lmx1b^Icst/+^* eyes are buphthalmic and display a variety of abnormalities, as previously described [18]. Examples are shown in Figure 2B and 2C. Strands of tissue extend between the iris and cornea in some mice (Figure 2B) and corneal neovascularisation is seen, often associated with corneal ulcers (Figure 2C). Other abnormalities in the cornea included scarring and inflammation, with wrinkling of Descemet's membrane and flattening of the endothelial cells with migration onto the anterior iris surface (Figure 2E). It is likely that endothelial abnormalities contributed to the development of corneal oedema (Figure 2F) and ocular surface compromise which in some cases led to ulceration. Many *Lmx1b^Icst/+^* eyes develop severe corneal ulcers with age. The bulging and distended appearance of the eyes suggested that there might be dysfunction of the drainage system at the iridocorneal angle resulting in raised intraocular pressure. We examined the angle by histology and found abnormalities that varied between eyes. In some *Lmx1b^Icst/+^* eyes there was an open angle (Figure 2H) but in other cases the angle was closed with the iris adhering to the cornea (Figure 2I and 2J). Although the angle is still open in some eyes, it is often narrow and sometimes Schlemm's canal appears abnormally short (Figure 2L). The trabecular meshwork, a structure dependent on *Lmx1b* for its development [16], typically appears abnormal, often being compressed and hypomorphic (Figure 2L). In line with these histological observations, we found that intraocular pressure (IOP) was elevated in *Lmx1b^Icst^* heterozygotes (Figure 3). Between the ages of two and six months IOP was elevated in *Lmx1b^Icst^* heterozygotes compared to wild-types (Figure 3A). The mean IOP measurements in older mice at 6–12 months are not statistically different between the two groups, in part due to an increased incidence of low IOP values (<10 mmHg) in older *Lmx1b^Icst^* heterozygotes, which is caused by significant corneal damage (ulceration, sometimes with inflammation and perforation). Overall, about 35% of *Lmx1b^Icst^* heterozygotes were found to have high IOP as compared to about 5% of wild-types (Figure 3B). Higher IOP is most commonly associated with poorly understood abnormalities of the iridocorneal angle [23] and it is a common and important risk factor for developing glaucoma in humans and can lead to the neurodegenerative hallmarks of the disease which are retinal ganglion cell loss and optic disc cupping. We observed optic nerve cupping in some, but not all, *Lmx1b^Icst^* heterozygous mice at various ages (Figure 4A). There was also a profound reduction in the number of retinal ganglion cells in *Lmx1b^Icst^* heterozygous mice that was not seen in *Lmx1b^KO^* heterozygous mice (Figure 4B). In addition, in *Lmx1b^Icst^* heterozygous mice optic nerve damage occurs and the loss of axons appear to be progressive (Figure 4C).

### *Lmx1b^Icst^* is a dominant semi-lethal allele of *Lmx1b*

**Figure 5:**
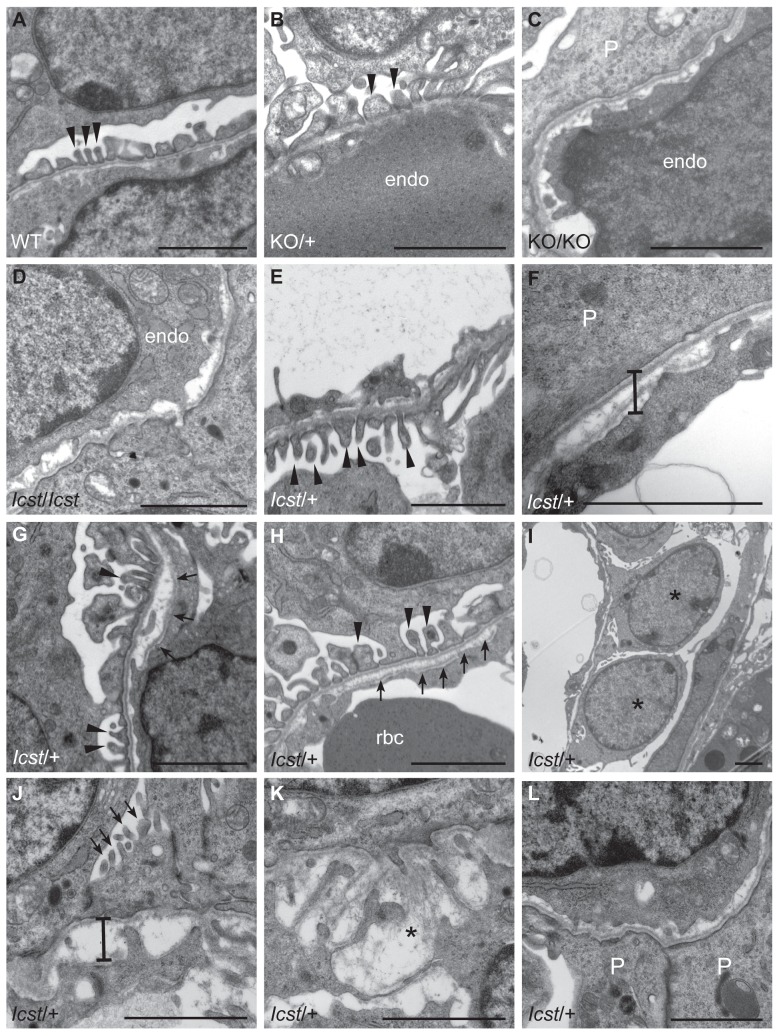
Ultrastructural analysis of E17.5 and E18.5 kidneys shows that *Icst* causes glomerular defects when heterozygous. (A–B) Wild-type (WT) and *Lmx1b^KO/+^* (KO/+) podocytes are normal with normal foot process formation (arrowheads). (C–D) *Lmx1b^KO/KO^* (KO/KO) and *Lmx1b^Icst/Icst^* (*Icst*/*Icst*) podocytes fail to form foot processes and appear immature. (E–L) *Lmx1b^Icst^* heterozygous podocytes (*Icst*/+). (E) Normal foot process formation (arrowheads). (F) The bar indicates an area of the GBM that appears fragmented. (G–H) Foot processes (arrowheads) abut GBM that is split (arrows). (I) The podocytes appear cuboidal (as indicated by asterisks) and immature. (J) The GBM is split as indicated by the bar and foot processes are rudimentary (arrows). (K) There is a large split in the GBM that is filled with fibrillar material (as indicated by an asterisk). (L) Podocytes are positioned flush against a split GBM. P, podocyte; endo = endothelial cell. Scale bar = 2 µm.

Homozygous knockout mice that lack LMX1B die on the day of birth [12]. They have ventralised limbs, absent cerebellum and kidney and eye defects [12], [14], [15]. We intercrossed heterozygous *Lmx1b^Icst^* mice and collected offspring for genotyping at E17.5 and at weaning. At E17.5 the three expected genotypes were present at Mendelian ratios indicating that *Lmx1b^Icst^* does not cause lethality *in utero* (Table 1). However, no *Lmx1b^Icst^* homozygous mice were present at weaning and four pups that were dead at birth were found to be homozygotes (Table 1). We examined homozygous mutant embryos and observed a phenotype very similar to that described for the knockout. *Lmx1b^Icst^* homozygotes lack a cerebellum, have abnormal kidneys and ventralised limbs (Figure S1 and Figure 5D). We expected this cross to produce twice as many *Lmx1b^Icst^* heterozygotes as wild-type. However, at weaning we found equal numbers of these genotypes, indicating a deficit of heterozygotes and suggesting that the *Icst* allele is semi-lethal (Table 1). We also crossed heterozygous *Lmx1b^Icst^* mice with mice heterozygous for the knockout allele *Lmx1b^tm1Rjo^* (hereafter, *Lmx1b^KO^*) and genotyped their offspring at weaning (Table 2). No double mutant mice were seen among 104 offspring, indicating that the two alleles do not complement each other and are thus allelic. There was a deficiency of about a third of *Lmx1b^Icst^* heterozygotes compared to *Lmx1b^KO^* heterozygotes, but this fell short of statistical significance (χ^2^ = 3.358, d.f. = 1, P = 0.067). To investigate this apparent dominant lethality further, we backcrossed both alleles of *Lmx1b* to C57BL/6J (Table 2) and genotyped the offspring. We found the expected 1∶1 ratio of heterozygotes to wild-type for the *Lmx1b^KO^* allele backcross whereas for the *Lmx1b^Icst^* allele a third fewer than expected heterozygotes were seen (χ^2^ = 26.3, d.f. = 1, P<0.0001). All of the mice carrying the *Icst* mutation had abnormal eyes showing that the mutant eye phenotype, as described in the previous section, is completely penetrant on the studied genetic backgrounds. The observed deficiency in the number of *Lmx1b^Icst^* heterozygotes indicates that the *Lmx1b^Icst^* allele is semi-lethal when heterozygous, in contrast the knockout allele which does not elicit a heterozygous phenotype. Therefore the mutant LMX1B^Icst^ protein must exert a dominant effect during development and/or early postnatal life that always results in ocular abnormalities and can be lethal.

**Table 1:** Genotyping results for *Lmx1b^Icst/+^* intercross.

Age	WT	*Icst*/+	*Icst*/*Icst*	Total	P^a^
adult	25	25	0	50	<0.0001
found dead at birth	1	1	4	6	0.059
E17.5	4	16	4	24	0.264

a Test for significance using Chi-square test.

**Table 2:** Genotyping results for backcross and intercross of the two *Lmx1b* alleles.

	Number of offspring at weaning				P^a^
Cross	WT	*Icst*/+	KO/+	KO/*Icst*	
*Lmx1b^Icst/+^*×wild-type	386	256	-	-	<0.0001
*Lmx1b^KO/+^*×wild-type	67	-	64	-	0.793
*Lmx1b^Icst/+^*×*Lmx1b^KO/+^*	37	26	41	0^b^	<0.0001

a Test for significance using Chi-square test.

b 4 pups found dead at birth with ventralised limbs.

- = not possible genotypes.

### *Lmx1b^Icst^* can cause glomerular defects when heterozygous

When and why do 30% of *Lmx1b^Icst^* heterozygous mice die? The deficiency of *Lmx1b^Icst/+^* mice at weaning (Table 2) was present at P1 (χ^2^ = 3.922, d.f. = 1, P = 0.048) (Table 3) suggesting the lethality is perinatal and any *Lmx1b^Icst^* ^/+^ mice that reach P1 survive as well as their wild-type littermates. We considered a possible cause of death to be kidney failure because of the importance of LMX1B in podocyte and slit diaphragm development [21], [24], [25]. We examined by electron microscopy the glomerular morphology of wild-type, *Lmx1b^Icst^* heterozygous and homozygous mice and, for comparison, glomeruli from *Lmx1b^KO^* heterozygous and homozygous mice at E17.5 and E18.5 (Figure 5). We took care to examine only the most mature glomeruli so that our analysis would not be confounded by comparing immature and mature podocytes. Both wild-type and *Lmx1b^KO^* heterozygous morphology was normal and no abnormalities were found (Figure 5A and 5B) whereas in the homozygous glomeruli the podocytes were effaced on the glomerular basement membrane (GBM) as previously reported (Figure 5C) [24], [25]. In *Lmx1b^Icst^* homozygotes, podocytes appeared undeveloped; the morphology resembling that seen in the knockout (Figure 5D). We also found glomerular abnormalities in all *Lmx1b^Icst^* ^/+^ embryos examined although the degree varied between individuals (Figure 5 E–L). In glomeruli from two *Lmx1b^Icst^* ^/+^ E17.5 embryos there was normal development in many areas with foot processes forming normally (Figure 5E). However, we found various abnormalities in both, representative examples are shown in Figure 5 F–I. In Figure 5F and 5H the GBM is split. In Figure 5F the podocyte is positioned flush against the GBM and foot processes have failed to develop and the GBM itself is fragmented, suggesting it is not adhering properly. In some glomeruli podocytes were immature and cuboidal in shape (Figure 5I). These two individuals might have been *Icst* heterozygotes which would have survived as the ultrastructural changes are not extensive. In another *Lmx1b^Icst^* heterozygote examined at E18.5 there was extremely abnormal morphology (Figure 5 J–L). The GBM is split (Figure 5J and 5K) and only some rudimentary foot process formation was observed (Figure 5J). Areas where podocytes were effaced onto a split GBM were also found (Figure 5L). As we found no evidence of normal glomerular development in this individual it is likely to be an example of one of the *Lmx1b^Icst^* heterozygotes which would die. This variability in the extent of the mutant kidney phenotype found in *Lmx1b^Icst^* heterozygotes is consistent with the finding that only one third of the *Lmx1b^Icst^* heterozygotes fail to survive.

**Table 3:** Survival of *Lmx1b^Icst/+^* compared to wild-type at P1 in an *Lmx1b^Icst/+^* x wild-type cross.

Age	WT	*Icst*/+	Total	P^b^
found dead at P0	7	18	25	0.028
found dead at P1	15	19	34	0.493
Live pups at P1^a^	61	41	102	0.048

a 67 live pups observed at P0 missing at P1 (unknown genotype).

b Test for significance using Chi-square test.

### Transgenic rescue of *Lmx1b^Icst^* and *Lmx1b^KO^* mutant phenotypes

We have shown above that *Lmx1b^Icst^* induces gain-of-function heterozygous phenotypes of the eye and the kidney that are not found in heterozygotes for the knockout allele. Both alleles are homozygous lethal at birth. To investigate if wild-type *Lmx1b* could rescue these phenotypes we made mice that were transgenic for a bacterial artificial chromosome, RP23-305A12, which contains the *Lmx1b* gene centrally located in a 225 kb insert (Tg(RP23-305A12), hereafter BAC). This transgene was introduced into both the *Lmx1b^Icst^* and *Lmx1b^KO^* lines and mice crossed to assess if it could rescue the mutant phenotypes. First we examined the effect of the transgene on the *Icst* heterozygous and homozygous phenotypes. When hemizygous, the transgene rescued the *Lmx1b^Icst^* heterozygous gross eye phenotype (Figure S2). However, the hemizygous transgene did not rescue the perinatal lethality affecting *Lmx1b^Icst^* homozygotes (Table 4). In contrast, the hemizygous transgene could rescue the perinatal lethality of the homozygous knockout allele, although the number of *Lmx1b^KO^* homozygous rescued pups was low and they were small in size with ventralised limbs (see below) (Table 4). Interestingly, variable rescue of the eye phenotype of transgenic *Lmx1b^KO^* homozygous mice was observed; although the majority of eyes were normal, some eyes were very abnormal, with damaged corneas and optic nerve heads (Figure S3).

**Table 4:** The transgenic BAC rescues the *Icst* heterozygous semi-lethal phenotype but not the homozygous lethal phenotype and can rescue the knockout homozygous lethal phenotype.

Cross			
*Lmx1b^Icst/+^*×*Lmx1b^Icst/+^* [BAC]		*Lmx1b^KO/+^*×*Lmx1b^KO/+^* [BAC]	
Genotype	Number at weaning	Genotype	Number at weaning
WT [BAC]	14	WT [BAC]	22
WT	17	WT	23
*Icst*/+ [BAC]	24	KO/+ [BAC]	32
*Icst*/+	19	KO/+	35
*Icst*/*Icst* [BAC]	0	KO/KO [BAC]	3^a^
*Icst*/*Icst*	0	KO/KO	0
P^b^	0.4129	P^b^	0.2123

a 11 pups that survived birth and had ventralised limbs died before weaning assumed to have this genotype.

b Test for significance using Chi-square test of the frequency of the first 4 genotypes.

We next asked if increasing the dose of transgenic *Lmx1b* could better rescue the mutant phenotypes (Table 5). We found that two copies of the transgene could rescue the *Lmx1b^Icst^* heterozygous semi-lethality; transgenic *Lmx1b^Icst^* heterozygotes survived in the normal Mendelian ratio. Two BAC copies could also rescue the homozygous perinatal lethality, albeit inefficiently (Table 5). The *Lmx1b^Icst^* ^/*Icst*^ rescued mice were smaller than their littermates and had the *Icst* mutant eye phenotype (Figure S4). For the knockout allele, a greater number of homozygous *Lmx1b^KO^* mice survived when the transgene was homozygous than when it was hemizygous (Table 5) and in most cases the eyes are grossly normal. Nevertheless, a few did have a mutant eye phenotype and in these cases expression of wild-type *Lmx1b* from the transgenic BAC was reduced (Figure S4).

**Table 5:** Homozygosity for the BAC transgene can rescue both the *Icst* and knockout lethal phenotypes.

Cross			
*Lmx1b^Icst/+^* [BAC×2] intercross		*Lmx1b^KO/+^* [BAC×2] intercross	
Genotype^a^	Number at weaning	Genotype^a^	Number at weaning
WT	22^b^	WT	35
*Icst*/+	44^c^	KO/+	78^e^
*Icst*/*Icst*	6^d^	KO/KO	24^f^
P^g^	0.0048	P^g^	0.1107

For each cross the progeny from two matings was counted.

a all are homozygous for the transgenic BAC.

b includes 1 taken for analysis before weaning.

c includes 3 taken for analysis before weaning. There were 2 found dead on P0.

d includes 5 taken for analysis before weaning. There were 4 that did not survive to weaning.

e includes 2 taken for analysis before weaning. There was 1 found dead on P1.

f includes 4 taken for analysis before weaning. There were 3 that that did not survive to weaning.

g Test for significance using Chi-square test.

**Figure 6:**
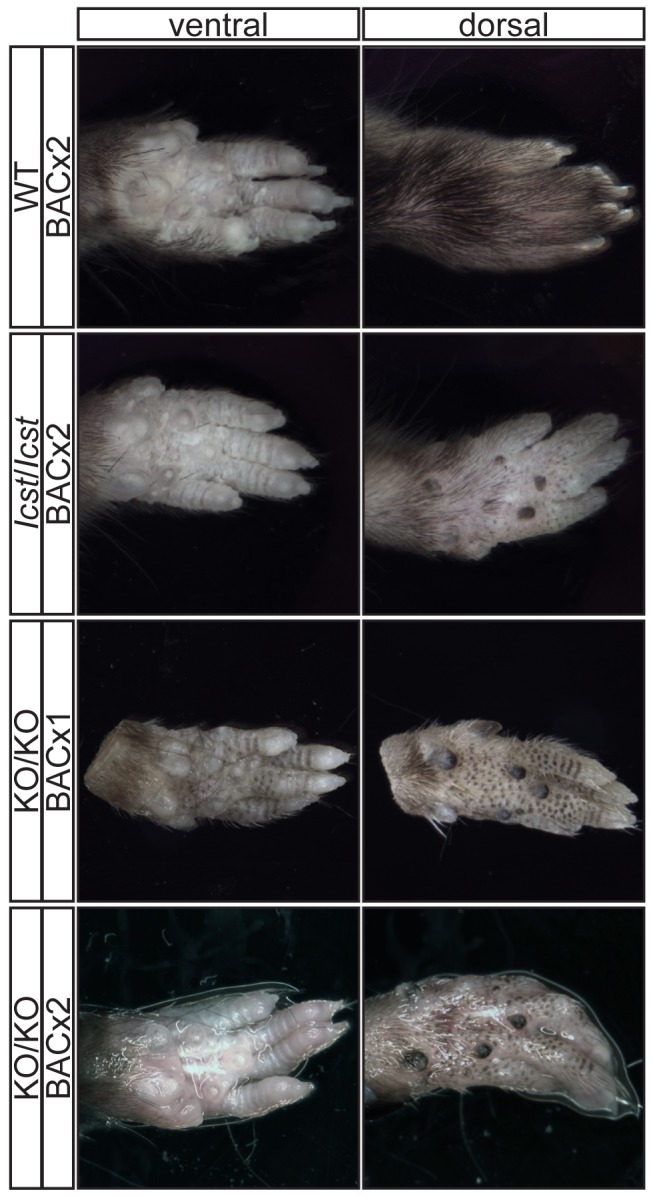
Forepaw phenotype of transgenic rescue mice. Pictures of the ventral and dorsal sides of forepaws from wild-type (WT), *Lmx1b^Icst^* ^/*Icst*^ (*Icst*/*Icst*) and *Lmx1b^KO/KO^* (KO/KO) mice are shown and whether the BAC transgene is hemizygous or homozygous is indicated (BACx1 or BACx2). The ventral side of the paw is normal for all the rescue mice. The dorsal surface of all the homozygous mutant paws appears ventralised with pigmented footpads and no hair. The ages of the mice shown are as follows. Top two rows, P14; third row P26 and bottom row P35.

**Figure 7:**
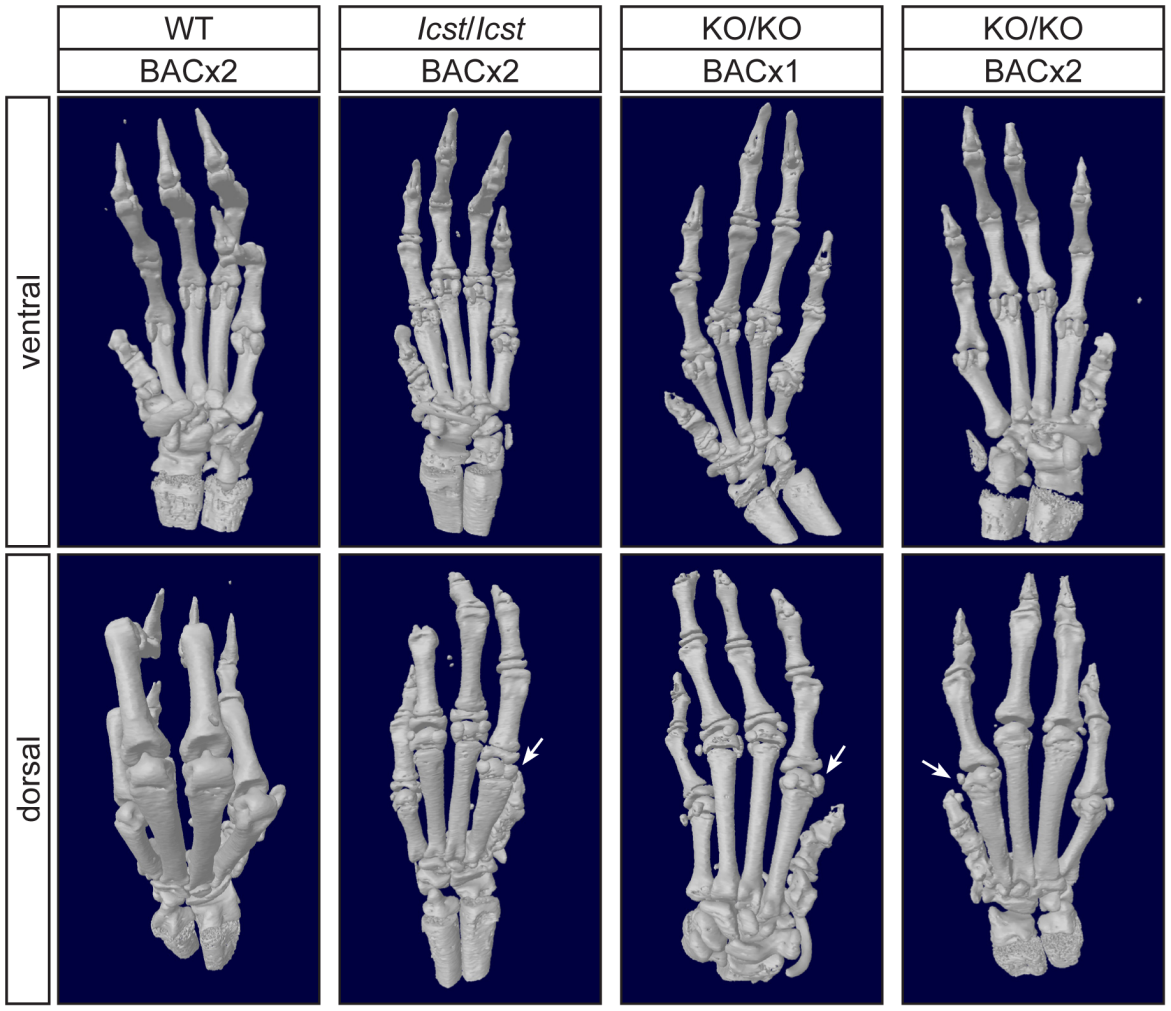
Forepaw skeletal phenotype of transgenic rescue mice. µCT scans of the ventral and dorsal sides from wild-type (WT), *Lmx1b^Icst^* ^/*Icst*^ (*Icst*/*Icst*) and *Lmx1b^KO/KO^* (KO/KO) forepaws are shown and whether the BAC transgene is hemizygous or homozygous is indicated (BACx1 or BACx2). The ventral side of the skeleton appears normal for all the rescue mice (top panels). The dorsal surface appears completely ventralised for the *Lmx1b^Icst^* ^/*Icst*^ rescue mice homozygous for the transgene and for the *Lmx1b^KO/KO^* rescue hemizygous for the BAC transgene. Arrows point to sesamoid bones that are a feature of the ventral surface. In the case of the *Lmx1b^KO/KO^* homozygous for the BAC transgene the dorsal surface appears more normal although there are still aspects of ventral morphology seen, for example a sesamoid bone (arrowed). The images shown are from mice between four and five weeks of age except for *Lmx1b^KO/KO^* hemizygous for the BAC transgene which was P26.

**Figure 8:**
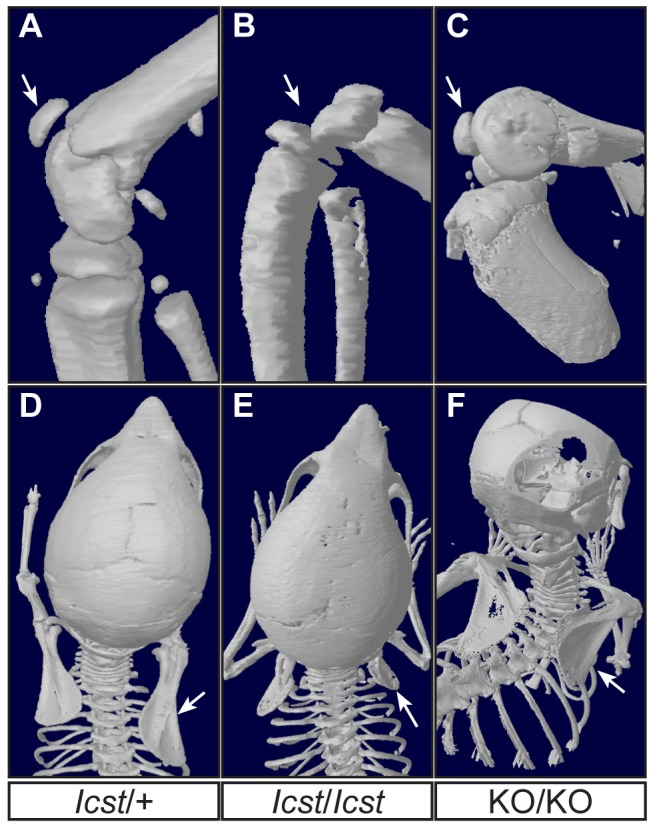
Patella and scapula phenotype of transgenic rescue mice. All mice shown are homozygous for the transgene. µCT scans of the patella and scapula are shown. (A) *Lmx1b^Icst^* ^/*+*^ (*Icst*/+) with normal patella (arrowed). (B) *Lmx1b^Icst^* ^/*Icst*^ rescue (*Icst*/*Icst*) knee where the patella is absent (arrow indicates its expected location). (C) *Lmx1b^KO/KO^* rescue (KO/KO) knee where the patella is present (arrowed). (D) Normal scapula in *Lmx1b^Icst^* ^/*+*^ (arrowed). (E) The scapula is very underdeveloped and small in *Lmx1b^Icst^* ^/*Icst*^ rescue (arrowed). (F) The scapula appears more normal in *Lmx1b^KO/KO^* rescue (arrowed). The ages of the mice shown are as follows; P23 (A, B, D, E), three months (C) and five weeks (F).

All the rescued mice, whether *Lmx1b^KO/KO^* or *Lmx1b^Icst/Icst^*, had skeletal and limb defects (Figures 6 and 7). *Lmx1b^Icst/Icst^* mice homozygous for the transgene had paws that were clearly ventralised; the dorsal surfaces were largely devoid of hair and had thickened skin pads superficially, much like the ventral surface, although the skin was pigmented (Figure 6). *Lmx1b^KO/KO^* mice rescued by a hemizygous BAC transgene had the same ventralisation phenotype which was not substantially improved when the transgene was homozygous (Figure 6). However, when we examined the skeletons of these homozygous transgenic rescue mice by X-ray computed microtomography (µCT), we found that aspects of the skeletal phenotype had been rescued (Figure 7). The paw skeleton of *Lmx1b^Icst/Icst^* mice homozygous for the BAC transgene was ventralised, as was that of *Lmx1b^KO/KO^* mice hemizygous for the BAC transgene (Figure 7). When homozygous the BAC transgene largely rescued the paw skeleton to near wild-type in *Lmx1b^KO/KO^* mice, despite the ventralised surface (Figure 7). Other skeletal abnormalities were also differentially rescued. Amongst other skeletal defects in homozygous *Lmx1b^KO^* mice the patella is absent and the scapula is very small [12]. We find the same defects in homozygous *Lmx1b^Icst^* embryos (Figure S1 and data not shown), and absence of patella is, of course, a cardinal feature of NPS in human patients [1]. *Lmx1b^Icst^* heterozygotes (and *Lmx1b^KO^* heterozygotes) do have patellae (data not shown). When homozygous, the BAC transgene does not rescue the patellar and scapular defects in *Lmx1b^Icst/Icst^* mice (Figure 8B and 8E) but does in *Lmx1b^KO/KO^* mice (Figure 8C and 8F), again demonstrating that mutant phenotypes can be rescued more readily from the null background than when LMX1B^Icst^ protein is present.

These BAC transgenic experiments demonstrate that a higher level of wild-type *Lmx1b* expression (i.e. two BAC copies) is required to elicit rescue of the *Lmx1b^Icst^* mutant phenotype than the *Lmx1b^KO^* mutant phenotype. This is consistent with an *Lmx1b^Icst^* pathogenic mechanism with the LMX1B^Icst^ protein exerting a dominant-negative effect on co-expressed wild-type protein.

### Mechanism of the dominant-negative effect of the *Lmx1b^Icst^* allele

**Figure 9:**
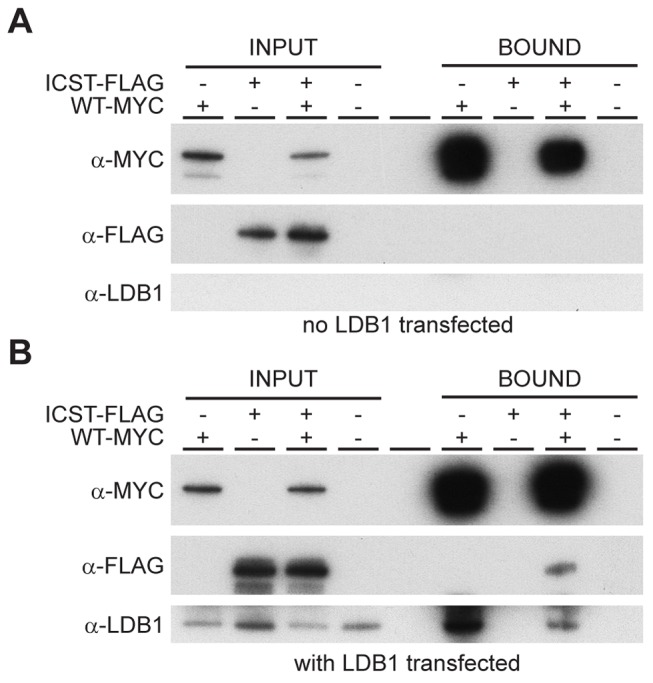
Wild-type and *Icst* LMX1B are in a complex with LDB1. Western blot analysis of co-immunoprecipitation experiments using anti-Myc (α-Myc), anti-FLAG (α-FLAG) and anti-LDB1 (α-LDB1) antibodies. (A) Protein lysates (INPUT) of HEK 293T cells transfected with WT-Myc and ICST-FLAG as indicated were immunoprecipitated using anti-c-Myc antibody coupled to agarose and the bound fraction (BOUND) eluted. WT-Myc is present in the immunoprecipitated bound fraction but Icst-FLAG is not. (B) Protein lysates (INPUT) of HEK 293T cells transfected with pcDNA-LDB1 along with WT-Myc and ICST-FLAG as indicated were immunoprecipitated using anti-c-Myc agarose and the bound fraction (BOUND) eluted. Both LDB1 and ICST-FLAG are in a complex with the WT-Myc and found in the immunoprecipitated bound fraction.

How does the LMXIB^Icst^ mutant protein exert this dominant-negative effect on the wild-type protein? LMX1B does not homodimerise [26], [27]. Along with other LIM-HD proteins, LMX1B binds to co-factors via the two LIM domains [28]. One such co-factor is LDB1 which can itself homodimerise thus enabling the formation of homomeric or heteromeric LIM-HD complexes [27]. This raises the possibility that complexes containing both wild-type and mutant LMX1B could be formed in *Lmx1b^Icst^* heterozygous mice thus decreasing the level of functional complexes below that found in *Lmx1b^KO^* heterozygous mice. To test if such complexes can be formed we transfected cells with Myc-tagged wild-type LMX1B and FLAG-tagged LMX1B^Icst^ either alone or together and immunoprecipitated using anti-Myc antibody. As expected the Myc-tagged wild-type LMX1B was present in the immunoprecipitated fraction but the FLAG-tagged LMX1B^Icst^ was not, confirming that LMX1B indeed does not homodimerise (Figure 9A). When LDB1 was included in the transfections, LDB1 was co-immunoprecipitated with the wild-type protein showing that LMX1B binds to LDB1 as expected (Figure 9B). When all three proteins were present, FLAG-tagged LMX1B^Icst^ protein was co-immunoprecipitated with the wild-type LMX1B (Figure 9B) showing that complexes containing both wild-type and mutant LMX1B are formed where the interaction is mediated by LDB1. Consistent with this, less LDB1 appears to be in the bound fraction when FLAG-tagged LMX1B^Icst^ was included along with the Myc-tagged wild-type LMX1B, probably due to competition between the wild-type and mutant protein for binding to LDB1 (Figure 9B).

## Discussion

### *Icst* is a dominant-negative mutation in *Lmx1b*

The *Icst* mutation, V265D, in the homeodomain of LMX1B abolishes DNA binding and transcriptional transactivation (Figure 1). Whilst heterozygous null (knockout allele) *Lmx1b* mice are phenotypically normal [12], [15] and present in Mendelian numbers (Table 2), a fraction of *Lmx1b^Icst^* heterozygous mice die with associated kidney GBM defects (Table 2 and Figure 5). No morphological abnormalities have been found in glomeruli of *Lmx1b* ^KO^ heterozygotes up to one year of age [24]. Those *Lmx1b^Icst^* heterozygotes that do survive have a highly penetrant eye phenotype which is not seen in *Lmx1b* ^KO^ heterozygotes (Figures 2–4). Depletion of *Lmx1b* expression in adult mice causes corneal opacity and neovascularisation indicating that a threshold level of *Lmx1b* expression in the cornea is necessary for the maintenance of corneal integrity [16]. These differences between the *Icst* and knockout heterozygous phenotypes indicate that the LMX1B^Icst^ protein exerts a dominant-negative effect on the wild-type protein. Dominant-negative mutant activity is typically mediated via protein complexes, usually dimers or higher-order structures, in which participation of one non-functional subunit inactivates the complex [29]–[33]. As previously reported [26], [27] and confirmed by our results (Figure 9) LMX1B does not homodimerise. We have shown that both wild-type LMX1B and LMX1B^Icst^ proteins are found in protein complexes mediated by LDB1 (Figure 9). Complexes containing both wild-type and Icst mutant protein are likely to be non-functional and in *Icst* heterozygotes the level of functional complexes containing only wild-type protein would be 25%, compared to 50% in the null heterozygotes. This provides an explanation for the difference in the heterozygous phenotype of the two alleles and leads to the prediction that missense mutation in the LIM domains abolishing protein-protein interactions would be equivalent to null alleles.

LMX1B has been shown to interact with LDB1 by yeast two-hybrid experiments [34] and complexes containing both proteins have been detected in rat glomeruli protein lysates [35]. The two genes have overlapping expression patterns [35], [36]. In support of the role of LDB1 in LMX1B function, in mice specific inactivation of *Ldb1* in podocytes leads to gradual loss of foot processes and GBM defects are found which lead to renal failure [35]. However, other binding partners for LMX1B are known, for example TCF3 [37], [38] raising the possibility that proteins other than LDB1 may be responsible for mediating the dominant-negative effect in some cell types.

### Differences in response to *LMX1B* mutations in humans and mice

There is a broad spectrum of disease severity both within and between NPS families and no clear genotype–phenotype correlation between the nature of mutations and severity of disease although in all cases skeletal abnormalities are found [5]. It is widely believed to be a haploinsufficient disorder and indeed, patients with a complete deletion of *LMX1B* have been found [6]. Variability in disease manifestation in patients with the same mutation is often observed [39] indicating genetic background modification. Furthermore, mutation of *LMX1B* does not always result in NPS. Two groups have reported the detection of novel missense mutations affecting R246 in the homeodomain of LMX1B in patients with isolated renal disease. In one report a patient with nail-patella-like renal disease was found to have an R246Q mutation that has residual transcription activity [40]. In the other report patients with autosomal dominant focal and segmental glomerular sclerosis either with the same R246Q mutation or with a different mutation affecting the same amino acid, R246P, were described [41]. None of the patients had any of the other classic symptoms of NPS. The reason why in these patients malformations are confined to the kidney is not clear. It may be that the residual activity of the R246Q protein is sufficient for normal development outwith the kidney or that these mutations of R246 only compromise binding to a subset of target sequences.

The lack of phenotype in *Lmx1b* null heterozygous mice [12], [15] contrasts with heterozygous *Lmx1b^Icst^* mice which have a strongly penetrant eye phenotype and have glomerular abnormalities that resemble defects found in human NPS patients (Figure 5). They do not, however, have any skeletal abnormalities, which is the most prevalent aspect of NPS. Valine 265 in LMX1B, the equivalent residue to that mutated in the mouse *Lmx1b^Icst^* allele, has been found mutated in NPS patients to phenylalanine and to leucine [8], [22]. V265L (originally reported as V242L) along with other patient missense mutations have been tested for dominant-negative effects on wild-type protein in *in vitro* transcription reporter assays but none have been found [7], [8]. Likewise, we have been unable to find, in *in vitro* experiments, a dominant-negative effect of the LMX1B^Icst^ protein on transcriptional reporter assays (data not shown), although it is clearly demonstrated by the phenotype in mice. It is apparent that mutant proteins can show dominant-negative activity in mice that is not seen *in vitro* and it is possible that some of the human *LMX1B* mutations may be dominant-negative. Indeed, it has been reported that patients with homeodomain mutations exhibit more severe proteinuria, and hence kidney defects, than patients with mutations in the LIM domains suggesting dominant-negative activity [9].

### *Icst* is a model for human glaucoma

Glaucoma is usually associated with high IOP caused by dysfunction of the ocular drainage structures in the iridocorneal angle of the eye [23]. *LMX1B* mutations are well established to cause open-angle glaucoma in NPS patients, but due to the influence of modifier genes may also cause glaucoma without NPS. Although confirmation is required, *LMX1B* haplotype has been suggested to influence open-angle glaucoma in the general population (without other aspects of NPS) [42]. Similar to some of the *Lmx1b^Icst^* heterozygous mice, a narrow but open-angle phenotype with high IOP is present in some individuals with an *LMX1B* mutation [4]. The aetiology of IOP elevation in glaucoma remains poorly understood and it is likely to be mechanistically heterogeneous at the molecular level. Various studies have reported open-angle glaucoma phenotypes in mice [43]–[47]. However, in most cases and due to undefined multifactorial influences, these phenotypes have typically been mild or have not yet proven reproducible between laboratories.

*Lmx1b^Icst/+^* mice have variably open or closed angles, and may be a model of anterior segment dysgenesis leading to high IOP, rather than primary open angle glaucoma. Nevertheless they should provide a valuable mouse model of glaucoma caused by dominant point mutation in a gene that also causes a form of human open-angle glaucoma. The phenotype is highly penetrant and reproducible in our colonies at different institutions. Thus, the *Icst* mutation provides a new tool for dissecting the molecular and pathologic features of IOP elevation and glaucoma, and for testing new therapies.

## Materials and Methods

### Ethics statement and animal husbandry

The animal studies described in this paper at the MRC Human Genetics Unit were carried out under the guidance issued by the Medical Research Council in *Responsibility in the Use of Animals for Medical Research* (July 1993) and Home Office Project Licence nos. PPL60/3124, PPL60/3785 and PPL60/4424. All experiments conducted at The Jackson Laboratory were approved by the institutional Animal Care and Use Committee. All animals were treated in accordance with the protocols established by the Association for Research in Vision and Ophthalmology. The Jackson Laboratory's pathogen surveillance program regularly screened for pathogens. Mice were housed in a 14 hour light to 10 hour dark cycle. Mutant and littermate control mice were housed together to control for cage-dependent differences. The *Lmx1b^Icst^* strain has been submitted to the European Mouse Mutant Archive (http://www. infrafrontier.eu) strain number EM:00114. Both the *Lmx1b^Icst^* and knockout lines were maintained on the C57BL/6J background. Clinical examinations were carried out as previously described [18], [48]. The BAC transgenic strain was made as described [49].

### Mutational analysis of *Lmx1b*

The exons and the immediate flanking sequences of *Lmx1b* were amplified from *Icst*, BALB/c, C3H and C57BL/6J genomic DNA using intronic primers that were also used for subsequent sequence analysis. PCR products were purified using Millipore Multi-screen PCR 96-well filtration system on a Biomek 2000 robotic platform and sequenced directly using Big Dye terminator cycle sequencing. Sequences were analysed using the Sequencher program.

### Constructs and recombinant protein production

To produce N-terminally histidine-tagged fusion proteins of full-length 372 amino acid LMX1B and the homeodomain alone, wild-type and *Icst* cDNAs were amplified by PCR introducing *Pci* I and *Nco* I sites at the 5′ of the full-length and homeodomain respectively and a *Bam* HI site at the 3′ of both and cloned into pGEM-T Easy (Promega). After digestion with *Bam* HI and *Pci* I or *Nco* I, as appropriate, the cDNAs were cloned into the *Nco* I and *Bam* HI sites of pET6H [50]. Recombinant histidine-tagged proteins were expressed in the *Escherichia coli* strain BL21 (DE3) pLysS essentially as described [51]. Proteins were analysed by sodium dodecyl sulphate–polyacrylamide gel electrophoresis to assess yield and purity and equal amounts of wild-type and mutant proteins were used in bandshift experiments. For expression in mammalian cells wild-type *Lmx1b* and *Lmx1b^Icst^* cDNAs were cloned into the expression vector pcDNA3.1 (Invitrogen). The original reported size of LMX1B protein is 372 amino acids [12]. An upstream in-frame ATG in the human sequence that would encode an additional 23 amino acids has been reported [22]. This sequence is conserved between mouse and human and the mouse LMX1B protein sequence in the database has been revised to include these extra 23 amino acids (entry O88609 in http://www.uniprot.org). In addition, there is a direct duplication of 18 bp encoding the first 6 amino acids of the 372 amino acid protein in the mouse genome sequence that is not conserved in human. This 18 bp sequence has been found to be absent from some *Lmx1b* cDNAs (e.g. BC125469) but it is found in the EST database (BY741174.1). By RT-PCR we found it to be present in *Lmx1b* transcripts (data not shown). We therefore made two versions of LMX1B, one 372 amino acids long (-S) and one including the additional N-terminal 29 amino acids (-L). Myc and FLAG-tagged mammalian expression vectors were made using the Gateway cloning system (Life Technologies). In brief, *Lmx1b^WT^* and *Lmx1b^Icst^* were amplified from the -L constructs described above and cloned into the donor vector pDONR™221 (Life Technologies) and then into the destination vectors pcDNA3.1Myc-HisDEST [52] and pDEST/C-SF-TAP [53] to give WT-Myc and Icst-FLAG respectively following the manufacturer's instructions. Full-length *Ldb1* was amplified from mouse embryonic cDNA using primers that introduced a Hind III site at the 5′ end and a Bam HI site at the 3′ end and cloned using these sites into pcDNA3.1 (+) to give pcDNA-LDB1. All plasmids were verified by sequencing.

### Bandshift analysis

The FLAT probe from the *Col4a4* gene intron 1 [21] was made by annealing the two oligonucleotides 5′-GGTTCATGAAAGTAATTATTTTCA-3′ and 5′-GGTTTGAAAATAATTACTTTCATG-3′ and end-labelled by filling in the four base 5′ single-stranded extensions with ^32^P dATP and ^32^P dCTP using Klenow polymerase. Bandshift analysis was carried out essentially as described [54]. 20,000 cpm FLAT probe (∼1×107 cpm/µg radiolabelled DNA probe) was used in each bandshift reaction. Bacterial extract protein concentrations were ∼5 µg/µl and we used 1, 2 or 3 µl per reaction.

### Transcription reporter assays

Transfections were carried out using a MicroPorator MP-100 following the manufacturer's protocol (Microporator). To correct for transfection efficiency and viability, 2.5 ng of renilla reporter vector was also transfected. The day following transfection luciferase assays were carried out using the Dual-luciferase reporter assay system (Promega E1910) and readings were normalised using the renilla reporter. Each experiment was carried out in triplicate.

### Mouse imaging

For anterior segment examination and photography, a Nikon FS-3V zoom slit-lamp biomicroscope was used with an attached Nikon D300S digital still camera and digital images were saved using Adobe Photoshop CS5 (Adobe, Inc.). Mouse paws were photographed using an imaging system comprising a Nikon AZ100 macroscope (Nikon UK Ltd, Kingston-on-Thames, UK) and a Qimaging Micropublisher 5 cooled colour camera (Qimaging, Burnaby, BC). Image capture was performed using in-house scripts written for IVision (BioVision Technologies, Exton, PA).

### Histology

Eye histology was carried out as previously described [18], [48]. After dissection embryos were photographed and fixed overnight in 4% paraformaldehyde in PBS at 4°C. A small part of the tail was used for genotyping. After washing in PBS they were dehydrated by immersion in a series of increasing concentrations of alcohol, embedded in paraffin wax, sectioned and stained with haematoxylin and eosin. Slides were viewed on a Leica MZFLIII fluorescence stereo microscope fitted with a Coolsnap colour camera (Roper Scientific, Tucson, Arizona, USA). Image capture was controlled by in-house scripting of IPLab Spectrum (Scanalytics, Fairfax, VA, USA). For plastic-based processing, enucleated eyes were fixed (0.8% paraformaldehyde and 1.2% glutaraldehyde in 0.08 M phosphate buffer (pH 7.4)) and processed for plastic sectioning as previously described [48]. Serial sagittal sections passing through the optic nerve were collected, stained with hematoxylin and eosin, and analysed for pathologic alterations.

### Intraocular pressure measurements

For all mice, IOP was measured as previously described in detail [55], [56]. Briefly, mice were acclimatised to the procedure room and anesthetized via an intraperitoneal injection of a mixture of ketamine (99 mg/kg; Ketalar, Parke-Davis, Paramus, NJ) and xylazine (9 mg/kg; Rompun, Phoenix Pharmaceutical, St. Joseph, MO) prior to IOP assessment.

### Ganglion cell counts

Eyes were collected into 2×PBS and fixed in 2% paraformaldehyde in PBS for two minutes. After rinsing in 2×PBS for five minutes the retina was dissected and laid flat by making radial incisions and fixed in methanol at −20°C for one hour. The retinas were then fixed again in 4% paraformaldehyde in PBS for five minutes, rinsed in 2×PBS and blocked in wholemount buffer (2×PBS, 1% Cohn fraction BSA, 3% Triton X-100) for one hour and then incubated with anti-BRN3 antibody (SC6026, Santa Cruz) overnight. After three ten minutes washes in wholemount buffer the retinas were incubated in Alexa Fluor 594 donkey anti-goat secondary antibody (1/500) (Molecular Probes) in wholemount buffer for four hours. After three washes in wholemount buffer, retinas were rinsed with 2×PBS and post-fixed in 4% paraformaldehyde in PBS for five minutes, mounted in Vectashield hard set (Vector Labs). All washes and incubations were carried out at room temperature on an orbital shaker. Four images were taken at 90° angles to each other around the optic disc using the Nikon TiE-C1Si confocal microscope. The number of BRN3-positive cells in each of the areas was counted using the Velocity Image acquisition and analysis software (PerkinElmer, Waltham, MA, USA). Mice from two litters were analysed. In the first litter (age four months) two *Lmx1b^icst^* ^/*+*^ mice were used and one each of the other two genotypes. In the second litter (age one month) one mouse of each genotype was used.

### Assessment of optic nerve damage

Optic nerves were processed and analysed as previously reported [48], [57]. Briefly, nerves were stained with paraphenylene-diamine which differentially stains single damaged axons allowing sensitive detection of axon injury. Nerves were determined to have one of three damage levels that are readily distinguishable by axon counting.

### Electron microscopy

E17.5 embryonic kidneys were fixed overnight in 3% glutaraldehyde in cacodylate buffer at 4°C then post-fixed in 1% osmium tetroxide for two hours at 4°C. After dehydration through ascending grades of alcohol and propylene oxide they were impregnated with TAAB Embedding Resin (medium grade premix) and cured for 24 hours. Ultrathin sections were stained with uranyl acetate and lead citrate and viewed on a JEOL JEM 100CXII fitted with an AMT Digital Camera using the AMTv600 image capture software.

### Skeletal imaging by µCT

Live animals and whole mount embryos and pups were scanned using a Skyscan 1076 *in vivo* µCT system (Skyscan B.V., Aartselaar, Belgium). Live animals were scanned under fluothane anaesthesia. Animals were scanned at an isotropic resolution of 18.6 µm. Scans were performed at 50 kV, 200 µA using a 0.5° rotation step and a 0.5 mm aluminium filter. Higher resolution scans of limbs were performed using a Skyscan 1172 system at a resolution of 8.8 µm (60 kV, 167 mA, 0.6° rotation step, 0.5 mm aluminium filter). Scans were reconstructed using Skyscan NRecon software and analysed using Skyscan CTAn software. Three dimensional models were visualised using Skyscan CTVol software.

### Co-immunoprecipitation

HEK 293T cells cultured in 100 mm dishes were transfected with WT-Myc (5 µg), ICST-FLAG (2 µg), and pcDNA-LDB1 (5 µg) as indicated in a total of 12 µg DNA adjusted with pcDNA3.1(-) as necessary using Lipofectamine LTX PLUS (Invitrogen). After 48 hours the cells were lysed using Cell Lysis Buffer (Cell Signaling Technology) and Myc-tagged complexes immunoprecipitated using the Profound c-Myc Tag IP/Co-IP Kit (Thermo Scientific) according to the manufacturer's instructions. Samples were separated on 4–12% NUPAGE gels (Life Technologies), transferred to Hybond-P (GE Healthcare) and probed with anti-Myc (Cell Signaling Technology, #2276), anti-FLAG (Cell Signaling Technology, #2368) and anti-LDB1(kind gift of Sam Pfaff [58]) antibodies using standard protocols and visualised with horseradish-peroxidase secondary antibody (GE Healthcare) and SuperSignal West Pico Chemiluminescent Substrate (Thermo Scientific) following the manufacturer's instructions.

### Statistical analysis

Values were expressed as mean+standard error and P<0.05 was considered significant. For the data shown in Figure 1 a two-tailed unpaired Student's *t*-test was used for statistical analysis. For the data shown in Figure 3 a two-tailed unequal variance *t*-test was performed using JMP (http://www.jmp.com). For the ganglion cell counts data shown in Figure 4 we used log transformation of raw data to allow for a mean-variance relationship. We then tested for a mean difference between replicate mice within genotype using ANOVA. As replicate mice within genotype differed significantly, we compared variation in mean cell count between pairs of genotypes against variation between means of replicate mice within genotype using a two-tailed Student's t-test. For the data shown in Tables 1–5 chi square tests were performed using http://graphpad.com/quickcalcs.

## Supporting Information

Figure S1 *Icst* homozygous phenotype. (A–B) µCT scans of E18.5 embryos. In the wild-type (WT) the frontal (F), paretial (P), interparietal (I) and occipital (O) bones are present. In *Lmx1b^Icst^* ^/*Icst*^ (*Icst*/*Icst*) the frontal, paretial and interparietal bones are fused (asterisk) and the occipital bone is missing (arrow). (C–D) Haematoxylin and eosin staining of sagittal brain sections at E16.5. The cerebellum is present in the wild-type (arrow) but absent in *Lmx1b^Icst^* ^/*Icst*^ where the inferior colliculus connects directly to the choroid plexus (asterisk). (E–F) Haematoxylin and eosin staining of pupil-optic nerve sections through embryonic eyes at E14.5. In *Lmx1b^Icst^* ^/*Icst*^ there is lens-corneal apposition. (G–H) Haematoxylin and eosin staining of sections through the paw at E18.5. Dorsal side is to the top. In the *Lmx1b^Icst^* ^/*Icst*^ paw there is duplication of the ventral muscle pattern. Scale bar = 500 µm. (I–J) Skeletal preparations of E18.5 hindlimbs. The patella is present in the wild-type (arrow) but absent from the *Lmx1b^Icst^* ^/*Icst*^ knee (arrow indicates its expected position).
(TIF)

Figure S2 Normal eye phenotype in *Lmx1b^Icst^* ^/*+*^ heterozygous for the transgenic BAC. Wild-type (WT) is shown on the left and *Lmx1b^Icst^* ^/*+*^ (*Icst*/+) is shown on the right. In both cases the mice are hemizygous for the transgene ([BAC]). The gross eye phenotype is shown in the top panels. Sections through the iridocorneal angle are shown in the middle panels and sections through the cornea are shown in the bottom panels. In all cases *Lmx1b^Icst^* ^/*+*^ mice with one copy of the transgene appear normal. Scale bar = 100 µm.
(TIF)

Figure S3 Eye phenotype of wild-type and homozygous knockout mice hemizygous for the transgenic BAC. Wild-type (WT) and *Lmx1b^KO/KO^* (KO/KO) hemizygous for the transgene ([BAC]) are shown. The external appearance, cornea and optic nerve head of the eye is normal in most homozygous knockout mice hemizygous for the transgenic BAC (middle panels) whereas in some mice abnormalities are seen (right panels). In this case the cornea has severe damage (middle panel, far right). Scale bar = 100 µm.
(TIF)

Figure S4 Eye phenotype of rescue mice homozygous for the transgenic BAC. (A) *Lmx1b^KO/KO^* (KO/KO) homozygous for the transgene ([BAC/BAC]) eye phenotype range from normal (top panels) to abnormal (middle and bottom panels). Sections through the cornea and lens are shown on the left and sections through the optic nerve are shown on the right. In the middle panels sections through the abnormal eye of an *Lmx1b^KO/KO^* with one abnormal eye are shown. The cornea appears thin and the optic nerve is normal. In the bottom panels sections through an abnormal eye of an *Lmx1b^KO/KO^* with two abnormal eyes are shown. The cornea is thickened and abnormal but the optic nerve appears normal. (B) *Lmx1b* expression from kidneys of *Lmx1b^KO/KO^* rescue mice with 0, 1 or 2 abnormal eyes. *Lmx1b* transcription is reduced in the rescued mutant mice with 1 or 2 phenotypically abnormal eyes compared to a rescued mutant mouse with normal eyes. Mean values and standard error of three independent quantitative RT-PCR reactions are shown. The expression of the sample with normal eyes was set to one and the relative expression of the other samples to this is shown. Experiment carried out as detailed in Protocol S1. (C) *Lmx1b^Icst^* ^/*Icst*^ rescue mice display the mutant eye phenotype in both eyes whereas BAC transgenic *Lmx1b^Icst^* ^/*+*^ have normal eyes. The transgene is homozygous ([BAC/BAC]). Sections through the cornea and lens are shown on the left and sections through the optic nerve are shown on the right. In the top left panel tissue that appears to be contiguous with the cornea is juxtaposed to the lens (arrowed) in *Lmx1b^Icst^* ^/*Icst*^ (*Icst*/*Icst*). The *Lmx1b^Icst^* ^/*+*^ (*Icst*/+) cornea shown in the bottom panel is normal. There is cupping of the optic nerve in *Lmx1b^Icst^* ^/*Icst*^ (arrowed) whereas the optic nerve of the *Lmx1b^Icst^* ^/*+*^ is normal. Scale bar = 100 µm.
(TIF)

Protocol S1 Real-time quantitative RT-PCR method.
(DOCX)

## References

[1] Sweeney E, Fryer A, Mountford R, Green A, McIntosh I (2003) Nail patella syndrome: a review of the phenotype aided by developmental biology. J Med Genet 40: 153–162. 12624132 10.1136/jmg.40.3.153 PMC1735400

[2] Mimiwati Z, Mackey DA, Craig JE, Mackinnon JR, Rait JL, et al (2006) Nail-patella syndrome and its association with glaucoma: a review of eight families. Br J Ophthalmol 90: 1505–1509. 16825280 10.1136/bjo.2006.092619 PMC1857543

[3] Dreyer SD, Zhou G, Baldini A, Winterpacht A, Zabel B, et al (1998) Mutations in LMX1B cause abnormal skeletal patterning and renal dysplasia in nail patella syndrome. Nat Genet 19: 47–50. 9590287 10.1038/ng0598-47

[4] Vollrath D, Jaramillo-Babb VL, Clough MV, McIntosh I, Scott KM, et al (1998) Loss-of-function mutations in the LIM-homeodomain gene, LMX1B, in nail-patella syndrome. Hum Mol Genet 7: 1091–1098. 9618165 10.1093/hmg/7.7.1091

[5] McIntosh I, Dreyer SD, Clough MV, Dunston JA, Eyaid W, et al (1998) Mutation analysis of LMX1B gene in nail-patella syndrome patients. Am J Hum Genet 63: 1651–1658. 9837817 10.1086/302165 PMC1377636

[6] Bongers EM, de Wijs IJ, Marcelis C, Hoefsloot LH, Knoers NV (2008) Identification of entire LMX1B gene deletions in nail patella syndrome: evidence for haploinsufficiency as the main pathogenic mechanism underlying dominant inheritance in man. Eur J Hum Genet 16: 1240–1244. 18414507 10.1038/ejhg.2008.83

[7] Dreyer SD, Morello R, German MS, Zabel B, Winterpacht A, et al (2000) LMX1B transactivation and expression in nail-patella syndrome. Hum Mol Genet 9: 1067–1074. 10767331 10.1093/hmg/9.7.1067

[8] Sato U, Kitanaka S, Sekine T, Takahashi S, Ashida A, et al (2005) Functional characterization of LMX1B mutations associated with nail-patella syndrome. Pediatr Res 57: 783–788. 15774843 10.1203/01.PDR.0000157674.63621.2C

[9] Bongers EM, Huysmans FT, Levtchenko E, de Rooy JW, Blickman JG, et al (2005) Genotype-phenotype studies in nail-patella syndrome show that LMX1B mutation location is involved in the risk of developing nephropathy. Eur J Hum Genet 13: 935–946. 15928687 10.1038/sj.ejhg.5201446

[10] Dunston JA, Lin S, Park JW, Malbroux M, McIntosh I (2005) Phenotype severity and genetic variation at the disease locus: an investigation of nail dysplasia in the nail patella syndrome. Ann Hum Genet 69: 1–8. 15638822 10.1046/j.1529-8817.2004.00133.x

[11] Dai JX, Johnson RL, Ding YQ (2009) Manifold functions of the Nail-Patella Syndrome gene Lmx1b in vertebrate development. Dev Growth Differ 51: 241–250. 19222527 10.1111/j.1440-169X.2008.01083.x

[12] Chen H, Lun Y, Ovchinnikov D, Kokubo H, Oberg KC, et al (1998) Limb and kidney defects in Lmx1b mutant mice suggest an involvement of LMX1B in human nail patella syndrome. Nat Genet 19: 51–55. 9590288 10.1038/ng0598-51

[13] Chen H, Ovchinnikov D, Pressman CL, Aulehla A, Lun Y, et al (1998) Multiple calvarial defects in lmx1b mutant mice. Dev Genet 22: 314–320. 9664684 10.1002/(SICI)1520-6408(1998)22:4<314::AID-DVG2>3.0.CO;2-9

[14] Guo C, Qiu HY, Huang Y, Chen H, Yang RQ, et al (2007) Lmx1b is essential for Fgf8 and Wnt1 expression in the isthmic organizer during tectum and cerebellum development in mice. Development 134: 317–325. 17166916 10.1242/dev.02745

[15] Pressman CL, Chen H, Johnson RL (2000) LMX1B, a LIM homeodomain class transcription factor, is necessary for normal development of multiple tissues in the anterior segment of the murine eye. Genesis 26: 15–25. 10660670

[16] Liu P, Johnson RL (2010) Lmx1b is required for murine trabecular meshwork formation and for maintenance of corneal transparency. Dev Dyn 239: 2161–2171. 20568247 10.1002/dvdy.22347 PMC5863528

[17] Endele S, Klein S, Richter S, Molter T, Amann K, et al (2007) Renal phenotype in heterozygous Lmx1b knockout mice (Lmx1b+/−) after unilateral nephrectomy. Transgenic Res 16: 723–729. 17657578 10.1007/s11248-007-9118-7

[18] Thaung C, West K, Clark BJ, McKie L, Morgan JE, et al (2002) Novel ENU-induced eye mutations in the mouse: models for human eye disease. Hum Mol Genet 11: 755–767. 11929848 10.1093/hmg/11.7.755

[19] Keane TM, Goodstadt L, Danecek P, White MA, Wong K, et al (2011) Mouse genomic variation and its effect on phenotypes and gene regulation. Nature 477: 289–294. 21921910 10.1038/nature10413 PMC3276836

[20] Wilson DS, Guenther B, Desplan C, Kuriyan J (1995) High resolution crystal structure of a paired (Pax) class cooperative homeodomain dimer on DNA. Cell 82: 709–719. 7671301 10.1016/0092-8674(95)90468-9

[21] Morello R, Zhou G, Dreyer SD, Harvey SJ, Ninomiya Y, et al (2001) Regulation of glomerular basement membrane collagen expression by LMX1B contributes to renal disease in nail patella syndrome. Nat Genet 27: 205–208. 11175791 10.1038/84853

[22] Dunston JA, Hamlington JD, Zaveri J, Sweeney E, Sibbring J, et al (2004) The human LMX1B gene: transcription unit, promoter, and pathogenic mutations. Genomics 84: 565–576. 15498463 10.1016/j.ygeno.2004.06.002

[23] Quigley HA (2011) Glaucoma. Lancet 377: 1367–1377. 21453963 10.1016/S0140-6736(10)61423-7

[24] Rohr C, Prestel J, Heidet L, Hosser H, Kriz W, et al (2002) The LIM-homeodomain transcription factor Lmx1b plays a crucial role in podocytes. J Clin Invest 109: 1073–1082. 11956245 10.1172/JCI13961 PMC150943

[25] Miner JH, Morello R, Andrews KL, Li C, Antignac C, et al (2002) Transcriptional induction of slit diaphragm genes by Lmx1b is required in podocyte differentiation. J Clin Invest 109: 1065–1072. 11956244 10.1172/JCI13954 PMC150942

[26] Jurata LW, Gill GN (1997) Functional analysis of the nuclear LIM domain interactor NLI. Mol Cell Biol 17: 5688–5698. 9315627 10.1128/mcb.17.10.5688 PMC232417

[27] Jurata LW, Pfaff SL, Gill GN (1998) The nuclear LIM domain interactor NLI mediates homo- and heterodimerization of LIM domain transcription factors. J Biol Chem 273: 3152–3157. 9452425 10.1074/jbc.273.6.3152

[28] Hobert O, Westphal H (2000) Functions of LIM-homeobox genes. Trends Genet 16: 75–83. 10652534 10.1016/s0168-9525(99)01883-1

[29] Milner J, Medcalf EA (1991) Cotranslation of activated mutant p53 with wild type drives the wild-type p53 protein into the mutant conformation. Cell 65: 765–774. 2040013 10.1016/0092-8674(91)90384-b

[30] Willis A, Jung EJ, Wakefield T, Chen X (2004) Mutant p53 exerts a dominant negative effect by preventing wild-type p53 from binding to the promoter of its target genes. Oncogene 23: 2330–2338. 14743206 10.1038/sj.onc.1207396

[31] Aramayo R, Sherman MB, Brownless K, Lurz R, Okorokov AL, et al (2011) Quaternary structure of the specific p53-DNA complex reveals the mechanism of p53 mutant dominance. Nucleic Acids Res 39: 8960–8971. 21764777 10.1093/nar/gkr386 PMC3203597

[32] Isoda K, Roth S, Nusslein-Volhard C (1992) The functional domains of the Drosophila morphogen dorsal: evidence from the analysis of mutants. Genes Dev 6: 619–630. 1559611 10.1101/gad.6.4.619

[33] Lee D, Cross SH, Strunk KE, Morgan JE, Bailey CL, et al (2004) Wa5 is a novel ENU-induced antimorphic allele of the epidermal growth factor receptor. Mamm Genome 15: 525–536. 15366372 10.1007/s00335-004-2384-2

[34] Marini M, Bongers EM, Cusano R, Di Duca M, Seri M, et al (2003) Confirmation of CLIM2/LMX1B interaction by yeast two-hybrid screening and analysis of its involvement in nail-patella syndrome. Int J Mol Med 12: 79–82. 12792813

[35] Suleiman H, Heudobler D, Raschta AS, Zhao Y, Zhao Q, et al (2007) The podocyte-specific inactivation of Lmx1b, Ldb1 and E2a yields new insight into a transcriptional network in podocytes. Dev Biol 304: 701–712. 17316599 10.1016/j.ydbio.2007.01.020

[36] Asbreuk CH, Vogelaar CF, Hellemons A, Smidt MP, Burbach JP (2002) CNS expression pattern of Lmx1b and coexpression with ptx genes suggest functional cooperativity in the development of forebrain motor control systems. Mol Cell Neurosci 21: 410–420. 12498783 10.1006/mcne.2002.1182

[37] German MS, Wang J, Chadwick RB, Rutter WJ (1992) Synergistic activation of the insulin gene by a LIM-homeo domain protein and a basic helix-loop-helix protein: building a functional insulin minienhancer complex. Genes Dev 6: 2165–2176. 1358758 10.1101/gad.6.11.2165

[38] Johnson JD, Zhang W, Rudnick A, Rutter WJ, German MS (1997) Transcriptional synergy between LIM-homeodomain proteins and basic helix-loop-helix proteins: the LIM2 domain determines specificity. Mol Cell Biol 17: 3488–3496. 9199284 10.1128/mcb.17.7.3488 PMC232202

[39] Ragge NK, Brown AG, Poloschek CM, Lorenz B, Henderson RA, et al (2005) Heterozygous mutations of OTX2 cause severe ocular malformations. Am J Hum Genet 76: 1008–1022. 15846561 10.1086/430721 PMC1196439

[40] Isojima T, Harita Y, Furuyama M, Sugawara N, Ishizuka K, et al (2013) LMX1B mutation with residual transcriptional activity as a cause of isolated glomerulopathy. Nephrol Dial Transplant 29: 81–88. 24042019 10.1093/ndt/gft359

[41] Boyer O, Woerner S, Yang F, Oakeley EJ, Linghu B, et al (2013) LMX1B mutations cause hereditary FSGS without extrarenal involvement. J Am Soc Nephrol 24: 1216–1222. 23687361 10.1681/ASN.2013020171 PMC3736714

[42] Park S, Jamshidi Y, Vaideanu D, Bitner-Glindzicz M, Fraser S, et al (2009) Genetic risk for primary open-angle glaucoma determined by LMX1B haplotypes. Invest Ophthalmol Vis Sci 50: 1522–1530. 18952915 10.1167/iovs.08-2483

[43] Mabuchi F, Aihara M, Mackey MR, Lindsey JD, Weinreb RN (2004) Regional optic nerve damage in experimental mouse glaucoma. Invest Ophthalmol Vis Sci 45: 4352–4358. 15557443 10.1167/iovs.04-0355

[44] Dai Y, Lindsey JD, Duong-Polk X, Nguyen D, Hofer A, et al (2009) Outflow facility in mice with a targeted type I collagen mutation. Invest Ophthalmol Vis Sci 50: 5749–5753. 19797236 10.1167/iovs.08-3367 PMC6335031

[45] Senatorov V, Malyukova I, Fariss R, Wawrousek EF, Swaminathan S, et al (2006) Expression of mutated mouse myocilin induces open-angle glaucoma in transgenic mice. J Neurosci 26: 11903–11914. 17108164 10.1523/JNEUROSCI.3020-06.2006 PMC6674879

[46] Zhou Y, Grinchuk O, Tomarev SI (2008) Transgenic mice expressing the Tyr437His mutant of human myocilin protein develop glaucoma. Invest Ophthalmol Vis Sci 49: 1932–1939. 18436825 10.1167/iovs.07-1339 PMC2809368

[47] Zode GS, Kuehn MH, Nishimura DY, Searby CC, Mohan K, et al (2011) Reduction of ER stress via a chemical chaperone prevents disease phenotypes in a mouse model of primary open angle glaucoma. J Clin Invest 121: 3542–3553. 21821918 10.1172/JCI58183 PMC3163970

[48] Smith RS, John SW, Nishina PM, Sundberg JP, editors (2002) Systematic Evaluation of the Mouse Eye: Anatomy, Pathology and Biomethods. Boca Raton: CRC Press.

[49] Healy E, Jordan SA, Budd PS, Suffolk R, Rees JL, et al (2001) Functional variation of MC1R alleles from red-haired individuals. Hum Mol Genet 10: 2397–2402. 11689486 10.1093/hmg/10.21.2397

[50] Hu CH, McStay B, Jeong SW, Reeder RH (1994) xUBF, an RNA polymerase I transcription factor, binds crossover DNA with low sequence specificity. Mol Cell Biol 14: 2871–2882. 8164649 10.1128/mcb.14.5.2871-2882.1994 PMC358655

[51] Studier FW, Rosenberg AH, Dunn JJ, Dubendorff JW (1990) Use of T7 RNA polymerase to direct expression of cloned genes. Methods Enzymol 185: 60–89. 2199796 10.1016/0076-6879(90)85008-c

[52] Crow YJ, Leitch A, Hayward BE, Garner A, Parmar R, et al (2006) Mutations in genes encoding ribonuclease H2 subunits cause Aicardi-Goutieres syndrome and mimic congenital viral brain infection. Nat Genet 38: 910–916. 16845400 10.1038/ng1842

[53] Gloeckner CJ, Boldt K, Schumacher A, Roepman R, Ueffing M (2007) A novel tandem affinity purification strategy for the efficient isolation and characterisation of native protein complexes. Proteomics 7: 4228–4234. 17979178 10.1002/pmic.200700038

[54] Cross SH, McKie L, West K, Coghill EL, Favor J, et al (2011) The Opdc missense mutation of Pax2 has a milder than loss-of-function phenotype. Hum Mol Genet 20: 223–234. 20943750 10.1093/hmg/ddq457 PMC3005898

[55] John SW, Hagaman JR, MacTaggart TE, Peng L, Smithes O (1997) Intraocular pressure in inbred mouse strains. Invest Ophthalmol Vis Sci 38: 249–253. 9008647

[56] Savinova OV, Sugiyama F, Martin JE, Tomarev SI, Paigen BJ, et al (2001) Intraocular pressure in genetically distinct mice: an update and strain survey. BMC Genet 2: 12. 11532192 10.1186/1471-2156-2-12 PMC48141

[57] Libby RT, Li Y, Savinova OV, Barter J, Smith RS, et al (2005) Susceptibility to neurodegeneration in a glaucoma is modified by Bax gene dosage. PLoS Genet 1: 17–26. 16103918 10.1371/journal.pgen.0010004 PMC1183523

[58] Jurata LW, Kenny DA, Gill GN (1996) Nuclear LIM interactor, a rhombotin and LIM homeodomain interacting protein, is expressed early in neuronal development. Proc Natl Acad Sci U S A 93: 11693–11698. 8876198 10.1073/pnas.93.21.11693 PMC38120

[59] Anderson MG, Libby RT, Mao M, Cosma IM, Wilson LA, et al (2006) Genetic context determines susceptibility to intraocular pressure elevation in a mouse pigmentary glaucoma. BMC Biol 4: 20. 16827931 10.1186/1741-7007-4-20 PMC1543659

