# Correction: A Dominant-Negative Mutation of Mouse *Lmx1b* Causes Glaucoma and Is Semi-lethal via LBD1-Mediated Dimerisation

**DOI:** 10.1371/journal.pgen.1004917

**Published:** 2014-12-05

**Authors:** The PLOS Genetics Staff

In the title “LBD1” should appear as “LDB1”. The correct title should read “A Dominant-Negative Mutation of Mouse *Lmx1b* Causes Glaucoma and Is Semi-lethal via LDB1-Mediated Dimerisation”

## References

[1] Cross SH, Macalinao DG, McKie L, Rose L, Kearney AL, et al (2014) A Dominant-Negative Mutation of Mouse Lmx1b Causes Glaucoma and Is Semi-lethal via LBD1-Mediated Dimerisation. PLoS Genet 10(5): e1004359 doi:10.1371/journal.pgen.1004359 24809698 10.1371/journal.pgen.1004359 PMC4014447

