# Supplementary figures and images for: A Dominant-Negative Mutation of Mouse Lmx1b Causes Glaucoma and Is Semi-lethal via LBD1-Mediated Dimerisation
Source: PLoS Genet. 2014 May 8;10(5):e1004359. doi: 10.1371/journal.pgen.1004359 (PMC4014447; doi:10.1371/journal.pgen.1004359)

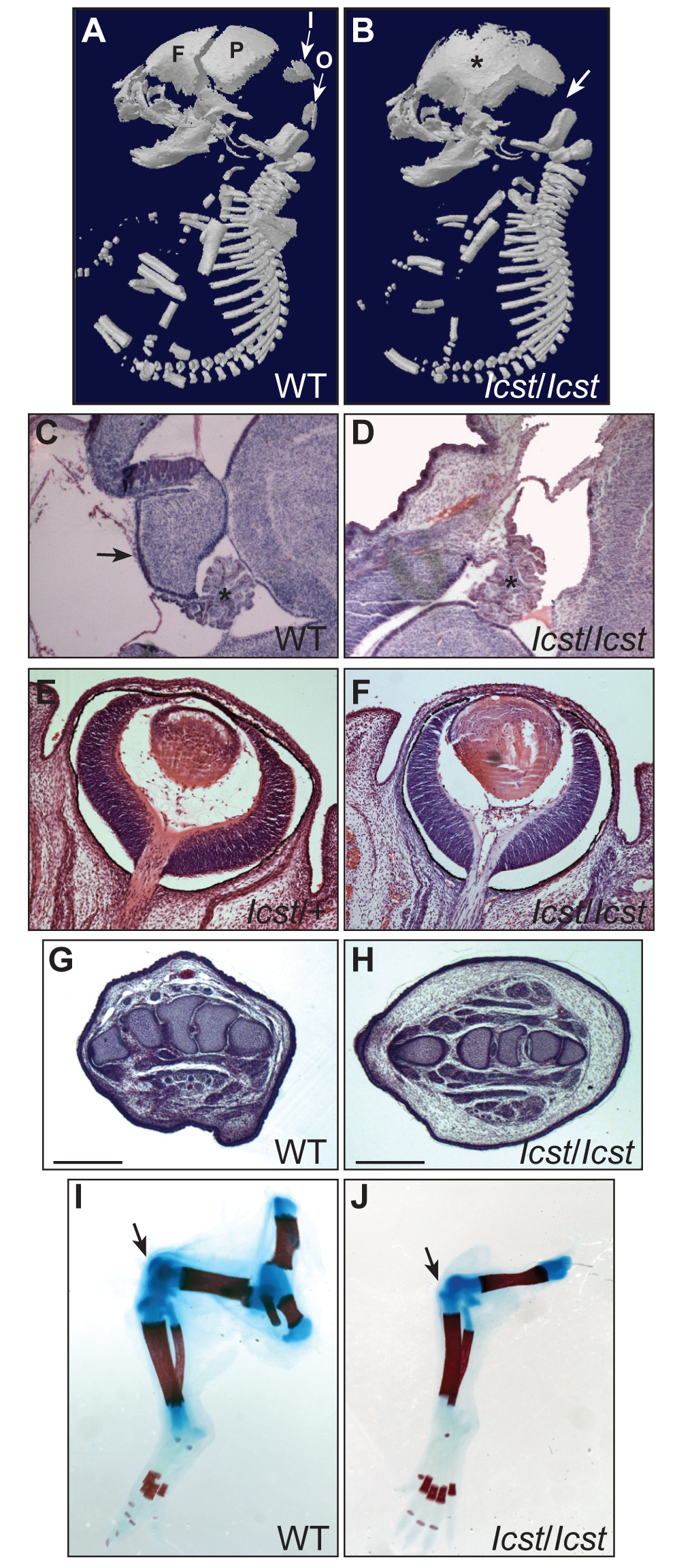

Supplement: Figure S1 — Icst homozygous phenotype. (A–B) µCT scans of E18.5 embryos. In the wild-type (WT) the frontal (F), paretial (P), interparietal (I) and occipital (O) bones are present. In Lmx1bIcst /Icst (Icst/Icst) the frontal, paretial and interparietal bones are fused (asterisk) and the occipital bone is missing (arrow). (C–D) Haematoxylin and eosin staining of sagittal brain sections at E16.5. The cerebellum is present in the wild-type (arrow) but absent in Lmx1bIcst /Icst where the inferior colliculus connects directly to the choroid plexus (asterisk). (E–F) Haematoxylin and eosin staining of pupil-optic nerve sections through embryonic eyes at E14.5. In Lmx1bIcst /Icst there is lens-corneal apposition. (G–H) Haematoxylin and eosin staining of sections through the paw at E18.5. Dorsal side is to the top. In the Lmx1bIcst /Icst paw there is duplication of the ventral muscle pattern. Scale bar = 500 µm. (I–J) Skeletal preparations of E18.5 hindlimbs. The patella is present in the wild-type (arrow) but absent from the Lmx1bIcst /Icst knee (arrow indicates its expected position). (TIF) [file pgen.1004359.s001.tif]

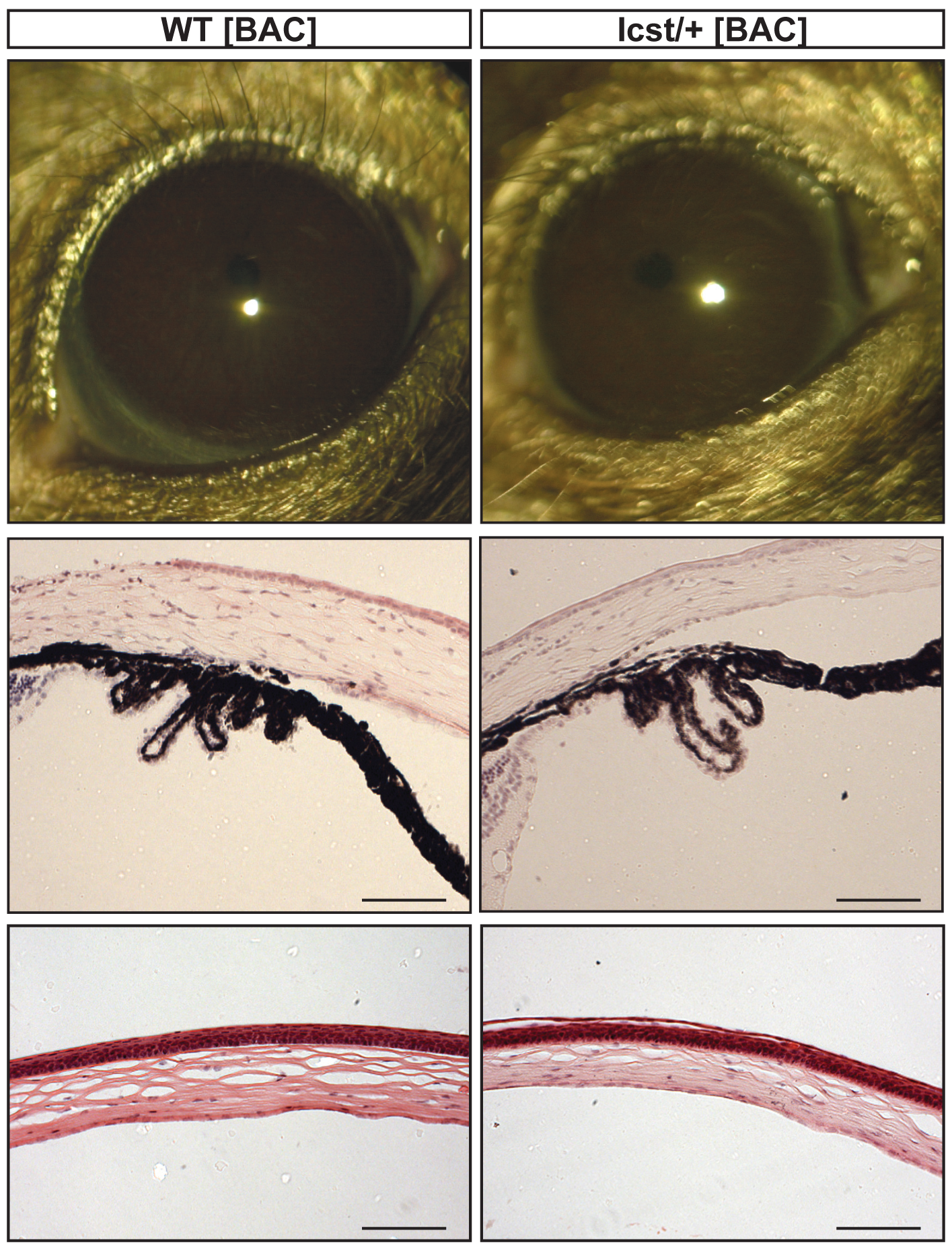

Supplement: Figure S2 — Normal eye phenotype in Lmx1bIcst /+ heterozygous for the transgenic BAC. Wild-type (WT) is shown on the left and Lmx1bIcst /+ (Icst/+) is shown on the right. In both cases the mice are hemizygous for the transgene ([BAC]). The gross eye phenotype is shown in the top panels. Sections through the iridocorneal angle are shown in the middle panels and sections through the cornea are shown in the bottom panels. In all cases Lmx1bIcst /+ mice with one copy of the transgene appear normal. Scale bar = 100 µm. (TIF) [file pgen.1004359.s002.tif]

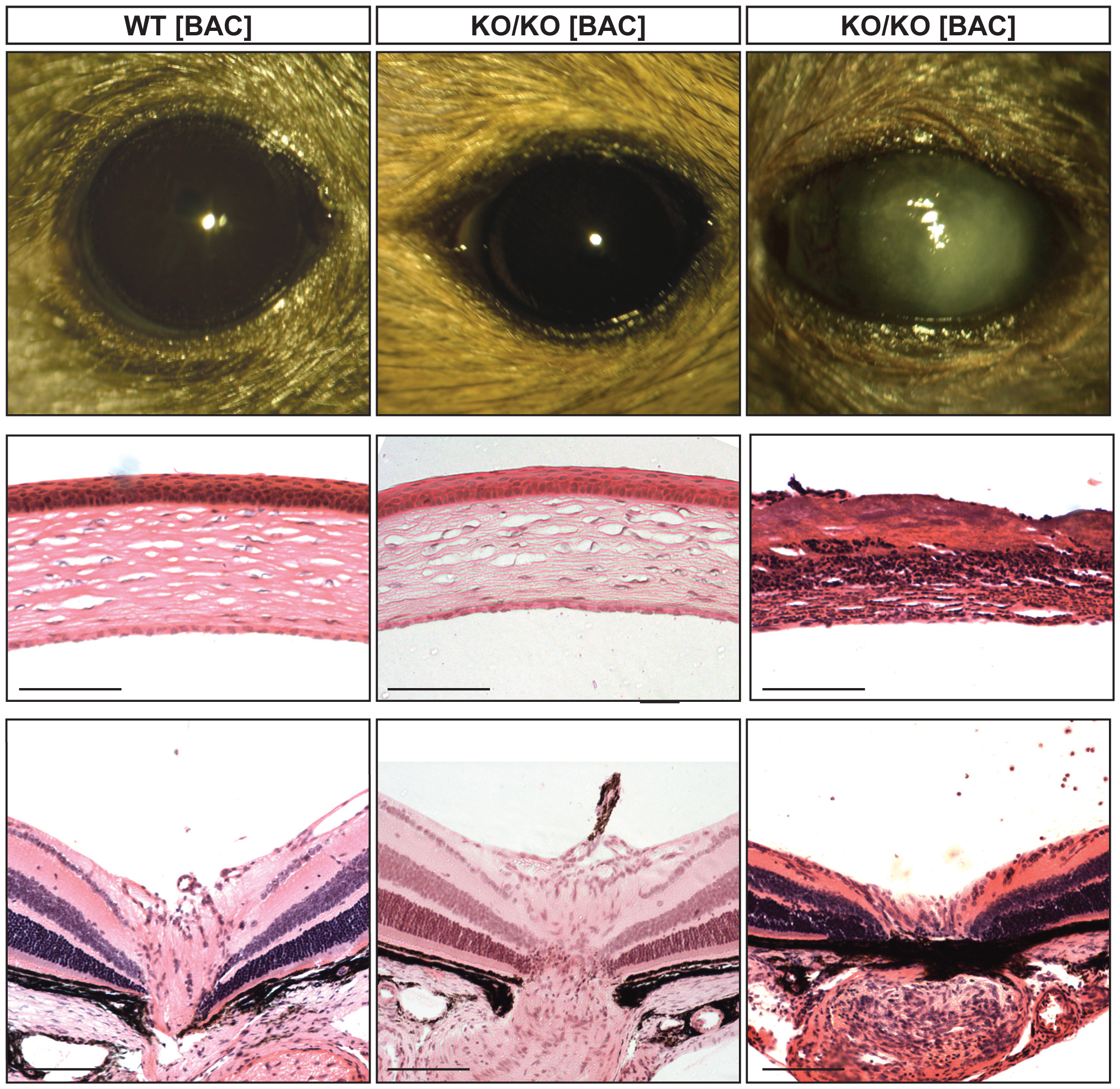

Supplement: Figure S3 — Eye phenotype of wild-type and homozygous knockout mice hemizygous for the transgenic BAC. Wild-type (WT) and Lmx1bKO/KO (KO/KO) hemizygous for the transgene ([BAC]) are shown. The external appearance, cornea and optic nerve head of the eye is normal in most homozygous knockout mice hemizygous for the transgenic BAC (middle panels) whereas in some mice abnormalities are seen (right panels). In this case the cornea has severe damage (middle panel, far right). Scale bar = 100 µm. (TIF) [file pgen.1004359.s003.tif]

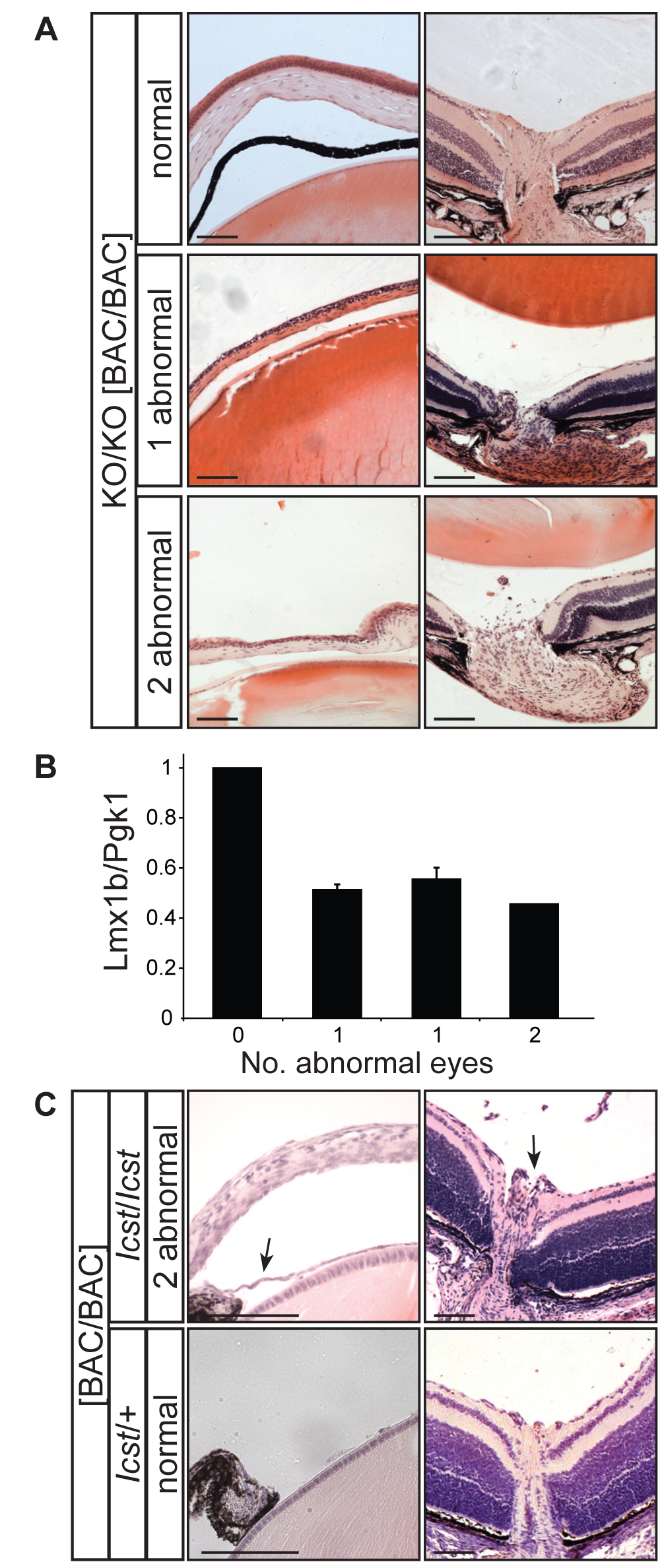

Supplement: Figure S4 — Eye phenotype of rescue mice homozygous for the transgenic BAC. (A) Lmx1bKO/KO (KO/KO) homozygous for the transgene ([BAC/BAC]) eye phenotype range from normal (top panels) to abnormal (middle and bottom panels). Sections through the cornea and lens are shown on the left and sections through the optic nerve are shown on the right. In the middle panels sections through the abnormal eye of an Lmx1bKO/KO with one abnormal eye are shown. The cornea appears thin and the optic nerve is normal. In the bottom panels sections through an abnormal eye of an Lmx1bKO/KO with two abnormal eyes are shown. The cornea is thickened and abnormal but the optic nerve appears normal. (B) Lmx1b expression from kidneys of Lmx1bKO/KO rescue mice with 0, 1 or 2 abnormal eyes. Lmx1b transcription is reduced in the rescued mutant mice with 1 or 2 phenotypically abnormal eyes compared to a rescued mutant mouse with normal eyes. Mean values and standard error of three independent quantitative RT-PCR reactions are shown. The expression of the sample with normal eyes was set to one and the relative expression of the other samples to this is shown. Experiment carried out as detailed in Protocol S1. (C) Lmx1bIcst /Icst rescue mice display the mutant eye phenotype in both eyes whereas BAC transgenic Lmx1bIcst /+ have normal eyes. The transgene is homozygous ([BAC/BAC]). Sections through the cornea and lens are shown on the left and sections through the optic nerve are shown on the right. In the top left panel tissue that appears to be contiguous with the cornea is juxtaposed to the lens (arrowed) in Lmx1bIcst /Icst (Icst/Icst). The Lmx1bIcst /+ (Icst/+) cornea shown in the bottom panel is normal. There is cupping of the optic nerve in Lmx1bIcst /Icst (arrowed) whereas the optic nerve of the Lmx1bIcst /+ is normal. Scale bar = 100 µm. (TIF) [file pgen.1004359.s004.tif]
