# Supplementary material for: A Dominant-Negative Mutation of Mouse Lmx1b Causes Glaucoma and Is Semi-lethal via LBD1-Mediated Dimerisation
Source: PLoS Genet. 2014 May 8;10(5):e1004359. doi: 10.1371/journal.pgen.1004359 (PMC4014447; doi:10.1371/journal.pgen.1004359)
Supplement: Protocol S1 — Real-time quantitative RT-PCR method. (DOCX) [file pgen.1004359.s005.docx]

**Protocol S1**

**Real-time quantitative RT-PCR method**

Quantitative RT–PCR of *Lmx1b* expression from adult kidney was carried out essentially as described using a Lightcycler 480 Instrument (Roche Diagnostics Ltd, Burgess Hill, West Sussex, UK) [1]. Each assay was done in triplicate and *Lmx1b* levels were quantified and normalized against *Pgk1* levels using the second derivative maximum method using the advanced relative quantification module of the Lightcycler 480 software release 1.5.0. *Pgk1* has been validated as a suitable reference gene for quantitative RT–PCR analysis of renal gene expression [2].

**References**

1. Cross SH, McKie L, West K, Coghill EL, Favor J, et al. (2011) The Opdc missense mutation of Pax2 has a milder than loss-of-function phenotype. Hum Mol Genet 20: 223-234.

2. Cui X, Zhou J, Qiu J, Johnson MR, Mrug M (2009) Validation of endogenous internal real-time PCR controls in renal tissues. Am J Nephrol 30: 413-417.
